# CRISPR/Cas9-mediated knock-in strategy at the *Rosa26* locus in cattle fetal fibroblasts

**DOI:** 10.1371/journal.pone.0276811

**Published:** 2022-11-28

**Authors:** Yuxuan Xie, Ming Wang, Liang Gu, Yang Wang

**Affiliations:** 1 Key Laboratory of Songliao Aquatic Environment, Ministry of Education, Jilin jianzhu University, Changchun, Jilin Province, People’s Republic of China; 2 College of Animal Science and Technology, China Agricultural University, Beijing, People’s Republic of China; Friedrich-Loeffler-Institute, GERMANY

## Abstract

The genetic modification of cattle has many agricultural and biomedical applications. However, random integration often leads to the unstable or differentially expression of the exogenous genes, which limit the application and development of transgenic technologies. Finding a safe locus suitable for site-specific insertion and efficient expression of exogenous genes is a good way to overcome these hurdles. In this study, we efficiently integrated three targeted vector into the cattle *Rosa26 (cRosa26)* by CRISPR/Cas9 technology in which EGFP was driven by *CAG*, *EF1a*, *PGK* and *cRosa26* endogenous promoter respectively. The CRISPR/Cas9 knock-in system allows highly efficient gene insertion of different expression units at the *cRosa26* locus. We also find that in the four cell lines, EGFP was stable expressed at different times, and the *CAG* promoter has the highest activity to activate the expression of EGFP, when compared with the *cRosa26*, *EF1a* and *PGK* promoter. Our results proved that *cRosa26* was a locus that could integrate different expression units efficiently, and supported the friendly expression of different expression units. Our findings described here will be useful for a variety of studies using cattle.

## Introduction

Genetically modified cattle hold great promise in the fields of agriculture and biomedicine, such as for improving the quality of milk [1], for producing the recombination of pharmaceutically active proteins [2, 3] and for improving resistance to zoonotic diseases [4, 5]. The traditional transgenic technique generally integrates exogenous genes into genome by random insertion. The integration site and the copy number cannot be accurately controlled, thus leading to the unstable or differentially expression of the exogenous genes, which limit the application and development of transgenic technologies. Site-specific integration maybe an effective way to overcome these hurdles [6].

In recent years, designer nucleases, such as zinc finger nucleases (ZFNs), transcription activator-like effector nucleases (TALENs) and clustered regularly interspaced short palindromic repeat (CRISPR)/CRISPR-associated 9 (Cas9), were reported to be suitable for genome editing in cattle [7–9]. Using these approaches, it is possible to integrate exogenous genes into a pre-selected locus with a single copy for comparative or subtractive gene function studies. Therefore, the selection of a “safe genomic acceptor site”, which is suitable for insertion and efficient expression of exogenous genes without perturbing the transcription of nearby endogenous genes, is of great importance [10].

*Rosa26* is the most widely used "safe locus" in mammalian genome, which was first discovered by Friedrich and Soriano in mice [11]. The study of *Rosa26* found that this loci can support the expression of exogenous genes at all stages of embryonic development and in all tissues of adults without adverse effects [12]. Now the targeted modification of *Rosa26* in mice has been widely used in continuous and conditional expression of exogenous genes. These modification result in the establishment of several hundred mouse models, which play important roles in the basic study such as gene function, disease models and drug development research [13–15]. After the discovery of *Rosa26* in mice, the *Rosa26* locus of human [16], rat [17], pig [18, 19], rabbit [20] and sheep [21] were continually determined through comparative study. This locus were highly conserved in sequence, and supported stable and high expression of exogenous genes in all species. Recently, the *cRosa26* locus has been characterized and targeted by a Cre-dependent reporter gene [22, 23]. However, whether this locus supports exogenous gene expression under exogenous promoter has not been verified.

To address this question, we constructed enhanced GFP (EGFP) reporter expression vectors driven by different promoters, *CAG*, *PGK* and *EF1α*, with the *cRosa26* endogenous promoter as a control, and efficiently integrated four targeted vectors into the *cRosa26* by CRISPR/Cas9 technology. We further conducted in vitro experiments to functionally validate its applicability. Our results may broad the use of safe locus *cRosa26* in cattle.

## Materials and methods

### Ethics statement

This study was approved by the Institutional Animal Care and Use Committee of the China Agricultural University under approval number SKLAB-2014-07-05.

### Construction of vectors

Vectors were constructed using standard molecular cloning methods. The *cRosa26*-PGK-NEO donor vector consisted of the 1.3 kb 5′ homology arm and the 1.2 kb 3′ homology arm flanking an neo gene and EGFP gene driven by *CAG*, *EF1a* and *PGK* promoter were inserted between the homologous arms. To construct CRISPR/Cas9 expression vectors, candidate sgRNAs were designed using online software programs, and each 20 bp target sequence was subcloned into the pX330 vector (Addgene 42230). All the vectors were confirmed through restriction digestion and Sanger sequencing.

### Screening of highly efficient sgRNA

The editing activity of each sgRNA was first assayed by using T7EI (New England Biolabs, USA) as described previously [24]. Briefly, 4 μg of each CRISPR/Cas9 expression vector was nucleofected into 1 × 10^6^ CFFs using Amaxa Nucleofector reagent (Lonza Group AG Basel, Switzerland) according to the manufacturer’s guidelines and the program T-016. After 72 h, the Cas9-treated cells were collected and the genomic DNA was extracted using a DNeasy Blood and Tissue kit (Qiagen, Hilden, Germany). PCR amplicons including nuclease target sites were generated using the primers: *Rosa26-F/Rosa26-R*, the primers are listed in Table 1. The 700 bp PCR amplicons were denatured by heating and annealed to form heteroduplex DNA using a thermocycler and then digested with T7E1 for 30 min at 37°C and then analyzed using agarose gel electrophoresis. Mutation frequencies (indels, %) were calculated by quantifying the relative using ImageJ software. Then the PCR amplicons of the most efficient sgRNA were further analyzed by TA-cloning and a DNA sequencing analysis to confirm the induction of CRISPR-mediated mutations at the expected site.

**Table 1:** List of primers used in the present study.

Primers	Sequence
ROSA26-F	GCCGCAATACCTTTATGGGAG
ROSA26-R	ATTGGTGGTGAAACCTGTCTG
P1	GGCAGCAGGACTCGAGTTAG
P2	TGGTGCAGATGAACTTCAGG
P3	TGAATGAACTGCAGGACGAG
P4	CCACATATCCAGGGCTCAAG
EGFP-F	GAACCGCATCGAGCTGAA
EGFP-R	TGCTTGTCGGCCATGATATAG
GAPDH-F	CATGTTTGTGATGGGCGTG
GAPDH-R	CATCGTGGAGGGACTTATGAC
SETD5-F	TGGATCCCATGTCAACCGTG
SETD5-R	TTCAGGGTCGTTGCCAGTAC
THUMPD3-F	AGGATCTTGGAAGCACTGCC
THUMPD3-R	TTCTGGGGCCACTTTCAGTC
LHFPL4-F	CTGCTTCGCCATCATCAACG
	GATGGTGCTGAAGTCGGTGA
LHFPL4-R	GATGGTGCTGAAGTCGGTGA
SRGAP3-F	TCTCTGACGCCTTCCAACAC
	CTGGTTGGCTCTCCTCTTGG
SRGAP3-R	CTGGTTGGCTCTCCTCTTGG

### Cell culture and transfection

The cell culture and transfection procedure was performed as previously described [22]. Briefly, primary CFFs were isolated from a Holstein cattle fetus by disaggregating the entire body, with the exception of the head and viscera, and cultured in Dulbecco’s Modified Eagle’s Medium (DMEM; Gibco, Grand Island, New York, USA) supplemented with 10% fetal bovine serum (FBS) (Gibco, Grand Island, New York, USA) at 37.5°C in an atmosphere of 5% CO_2_ and humidified air. Next, 4 μg of CRISPR/Cas9 expression vector and 4 μg of linearized donor vector were nucleofected into 1 × 10^6^ CFFs using Amaxa Nucleofector reagent (Lonza Group AG Basel, Switzerland) according to the manufacturer’s guidelines and the program T-016. G418 (1 mg/ml) selection was used in cell colonies that formed within 48 h after transfection, and the cell density was approximately 1 × 10^5^ cells/dish (10 cm). Individual cell clones were isolated 7–10 days after G418 selection, and then were expanded and cultured. Finally, for each individual cell clones one-third of them were used for extracting the genomic DNA and analysis of integration, and two-thirds of them were frozen for further analysis.

### Identification of positive cell clones by PCR

To identify positive cell clones, genomic DNA was extracted from a single cell clone using a DNeasy Blood and Tissue kit (Qiagen). To confirm the successful generation of targeted clones, two pairs of primers located between the donor and outside of the 5’ or 3’ homologous arm were used: the P1/P2 primer pair was used for the 5’ arm, and the P3/P4 primer pair was used for the 3’ arm. For the successful production of *Rosa26*-CAG-EGFP-targeted cells, the expected amplicon were 3.6 kb and 2.4 kb; For the successful production of *Rosa26*-PGK-EGFP-targeted cells, the expected amplicon were 2.4 kb and 2.4 kb; For the successful production of *Rosa26*-EF1a-EGFP-targeted cells, the expected amplicon were 2.5 kb and 2.4 kb; For the successful production of *Rosa26*-EGFP-targeted cells, the expected amplicon were 2.0 kb and 2.4 kb. The PCR procedure was performed using LA-Taq (Takara), with initial DNA denaturation at 94°C for 5 min, followed by 35 cycles of 94°C for 30 s, 60°C for 30 s and 72°C for 2–4 min and a final 10-min extension. The PCR products were sequenced by TA cloning. All the primers are listed in Table 1.

### Q-PCR analyses

The Q-PCR was performed as previously described [22]. Briefly, total mRNAs were extracted from various targeted cells using Trizol Reagent (Thermo Fisher Scientific, Waltham, MA, USA) prior to performing Q-PCR analyses. The cDNA templates were synthesized using a FastQuant RT Kit (Tiangen, China), and the genomic DNA was then digested with DNase I. Primers EGFP-F/ EGFP-R were used to detect EGFP. Primers SETD5-F/SETD5-R were used to detect SETD5. Primers THUMPD3-F/THUMPD3-R were used to detect THUMPD3. Primers LHFPL4-F/ LHFPL4-R were used to detect LHFPL4. Primers SRGAP3-F/SRGAP3-R were used to detect SRGAP3. Primers GAPDH-F/GAPDH-R were used to amplify cattle GAPDH as the reference. Q- PCR was performed using SYBR Premix Ex Taq (TaKaRa, RR820A) and the 7500 Real-Time PCR System (Applied systems), with the following parameters: 95°C for 30 s, followed by 40 two-step cycles at 95°C for 5 s and at 60°C for 4 s. The relative expression level of each gene was calculated by the ΔΔCt method, normalized to GAPDH expression.

### Western blotting

The Western blotting was performed as previously described [22]. Briefly, samples were isolated from targeted cells and wild-type (WT) cells and homogenized in cell lysis buffer for Western and IP analyses (Beyotime, Shanghai, China). After centrifugation at 10000 g for 10 min at 4°C, the total protein supernatants were collected, and protein concentrations were measured using a BCA Protein Assay kit (Beyotime, Shanghai, China). Approximately 20 μg of protein was separated on 10% SDS-PAGE gels and transferred to Immobilon-P membranes (MilliporeSigma, Burlington, MA, USA). After blocking in 3% BSA in TBST for 1 h, membranes were incubated with a EGFP antibody (dilution, 1:10000; Abcam, Cambridge, MA, USA) or cattle GAPDH antibody (dilution, 1:10000; Abcam, Cambridge, MA, USA) overnight at 4°C. After washes with TBST, membranes were incubated with a goat anti-rabbit antibody conjugated with horseradish peroxidase (dilution, 1:20000; Sino-American Co, Beijing, China) for 1 h followed by three washes with TBST. Protein signals were detected using an ECL Chemiluminescence kit (Thermo Fisher Scientific, Waltham, MA, USA).

## Results

### Screening of sgRNA with high cleavage efficiency to cRosa26

**Fig 1:**
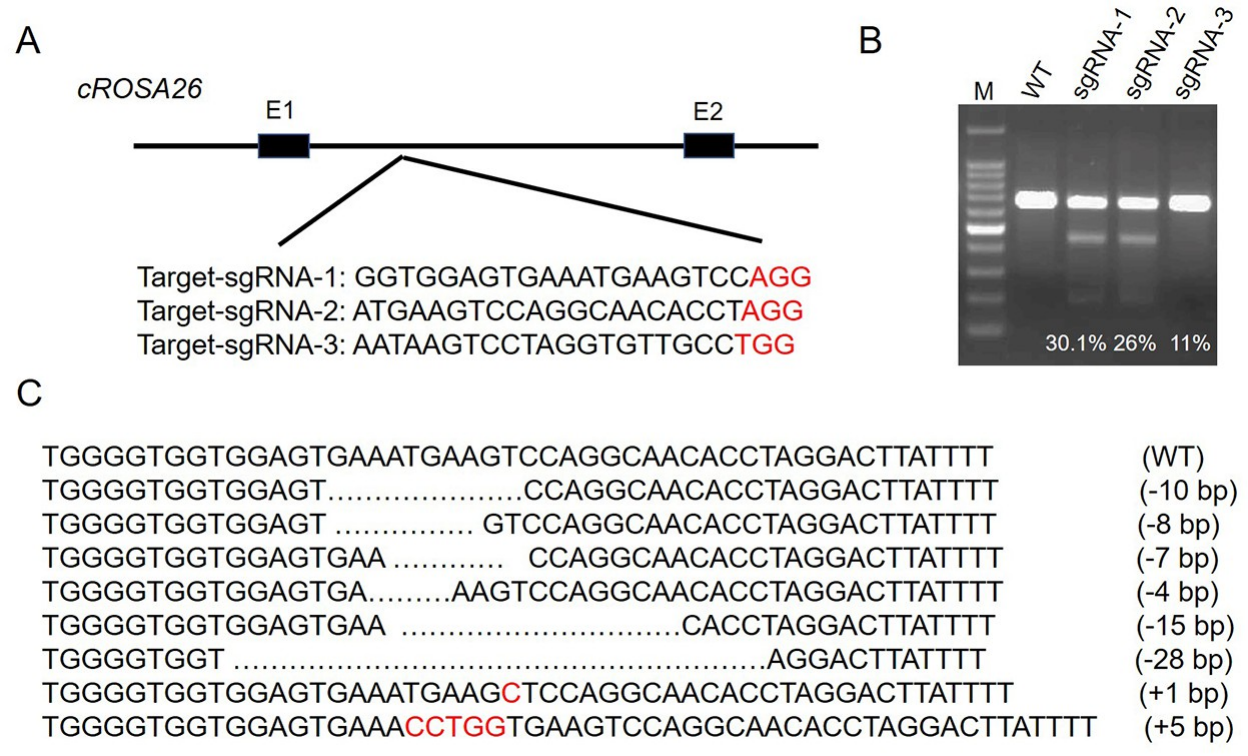
CRISPR/Cas9 induced targeted mutations at the *cRosa26* locus. (A) Modification of the *cRosa26* locus by CRISPR/Cas9. The CRISPR/Cas9 target sequences (20-bp target and 3-bp PAM sequence (colored in red)) are shown. (B) Representative results of T7EI assays. The mutation frequencies (% indels) of different sgRNAs were calculated by measuring the band intensities. M, 100-bp DNA ladder; WT, wild-type control cells; sgRNA-1, sgRNA-1-transfected cells; sgRNA-2, sgRNA-2-transfected cells; sgRNA-3, sgRNA-3-transfected cells. (C) Representative sequencing results of the TA clones revealing different indel mutations mediated by sgRNA-1 in the target site.

In this study, we selected the intron of the *cRosa26* locus for targeting and designed 3 guide RNAs (sgRNAs) (Fig 1A). To evaluate the transfection efficiency of CRISPR-targeting plasmids (sgRNA 1–3) in cattle fetal fibroblasts (CFFs), T7 endonuclease 1 (T7E1) assay was used. The CRISPR-targeting plasmids were transfected into the CFFs by electroporation. After 72 h, the genome of the CFFs was extracted and analyzed. sgRNA-1 cleaved the target site with the greatest efficiency, as evidenced by the increased incidence of allelic mutations (non-homologous end joining (NHEJ) frequency) (Fig 1B). TA-cloning and a DNA sequencing analysis of the PCR amplicons corroborated the induction of CRISPR-mediated mutations at the endogenous site (Fig 1C). Therefore, we used CRISPR-sgRNA-1 in subsequent experiments.

### Efficient CRISPR/Cas9-mediated site-specific integration into the cRosa26 locus

**Fig 2:**
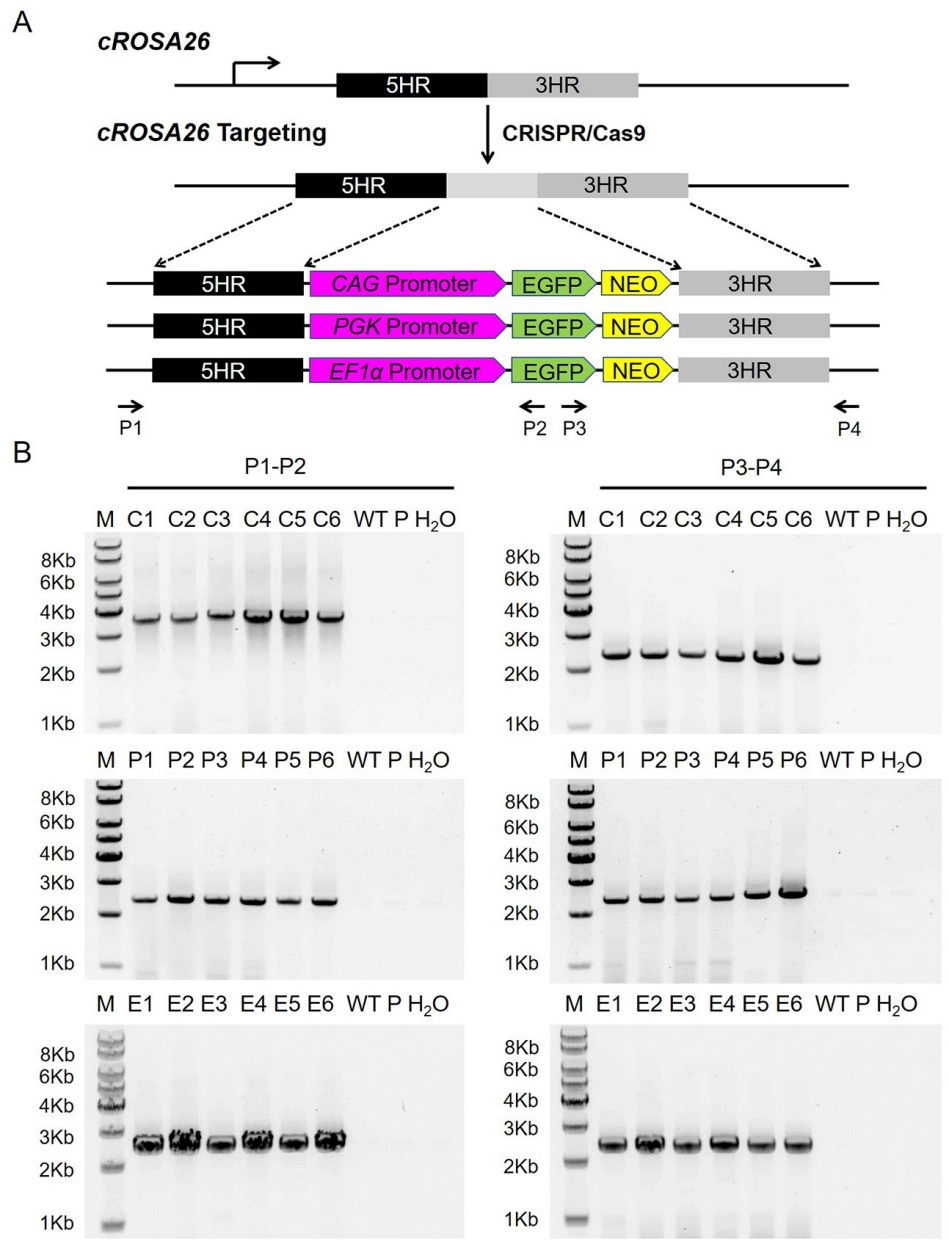
CRISPR/Cas9-mediated targeted site-specific integration at the *cRosa26* locus. (A) Schematic diagram of the *cRosa26* HDR template vector and strategy for insertion of various EGFP expression cassette into the *cRosa26* locus. EGFP, enhanced green fluorescent protein gene; NEO, neomycin-resistance gene; EGFP driven by *CAG*, *PGK*, and *EF1α* promoter, respectively. (B) PCR analysis of knock-in cell lines using the primer sets shown in A. M, 1 kb DNA ladder; C1-C6, the positive targeted integration of *CAG*-EGFP cassettes cell clones; P1-P6, the positive targeted integration of *PGK*-EGFP cassettes cell clones; E1-E6, the positive targeted integration of *EF1α*-EGFP cassettes cell clones; WT, wild-type cFFs; P, the donor vector; H_2_O was the negative control.

To check whether the *cRosa26* locus allowed widely and high activity of ubiquitous promoter, we chosen three ubiquitous promoters, *CAG*, *EF1a* and *PGK*, which were used commonly in genetically modified cattle, with the *cRosa26* endogenous promoter as a control, to drive EGFP into the *cRosa26* locus by traditional HR strategy (Fig 2A and S1 Fig). CFFs were electroporated with the linearized targeting vector accompanied by CRISPR-sgRNA-1. After selection with G418 (1 mg/ml from day 7 to day 10), 47 *EF1a*-EGFP, 48 *CAG*-EGFP, 50 *PGK*-EGFP, 28 EGFP (*cRosa26*-EGFP) cell clones were screened and expanded, respectively. As shown in Table 2, based on PCR analyses of the 5′- and 3′-arms, 39 of the 47 clones (*EF1a*-EGFP), 42 of the 48 clones (*CAG*-EGFP), 39 of the 50 clones (*PGK*-EGFP) and 24 of the 28 clones (*cRosa26-*EGFP) were correctly targeted. The PCR results of several positive clones are shown in Fig 2B and S1 Fig.

**Table 2:** Summary of the PCR results of G418-resistant targeted clones.

Targeting Vector	Screening method	Isolated colonies	positive cell colonies	Targeting efficiency (%)
Targeting-*EF1a*-EGFP	G418	47	39	82.9 (42/47)
Targeting-*CAG*-EGFP	G418	48	42	87.5 (42/48)
Targeting-*PGK*-EGFP	G418	50	39	78.0 (39/50)
Targeting- EGFP	G418	28	24	85.7 (24/28)

**Fig 3:**
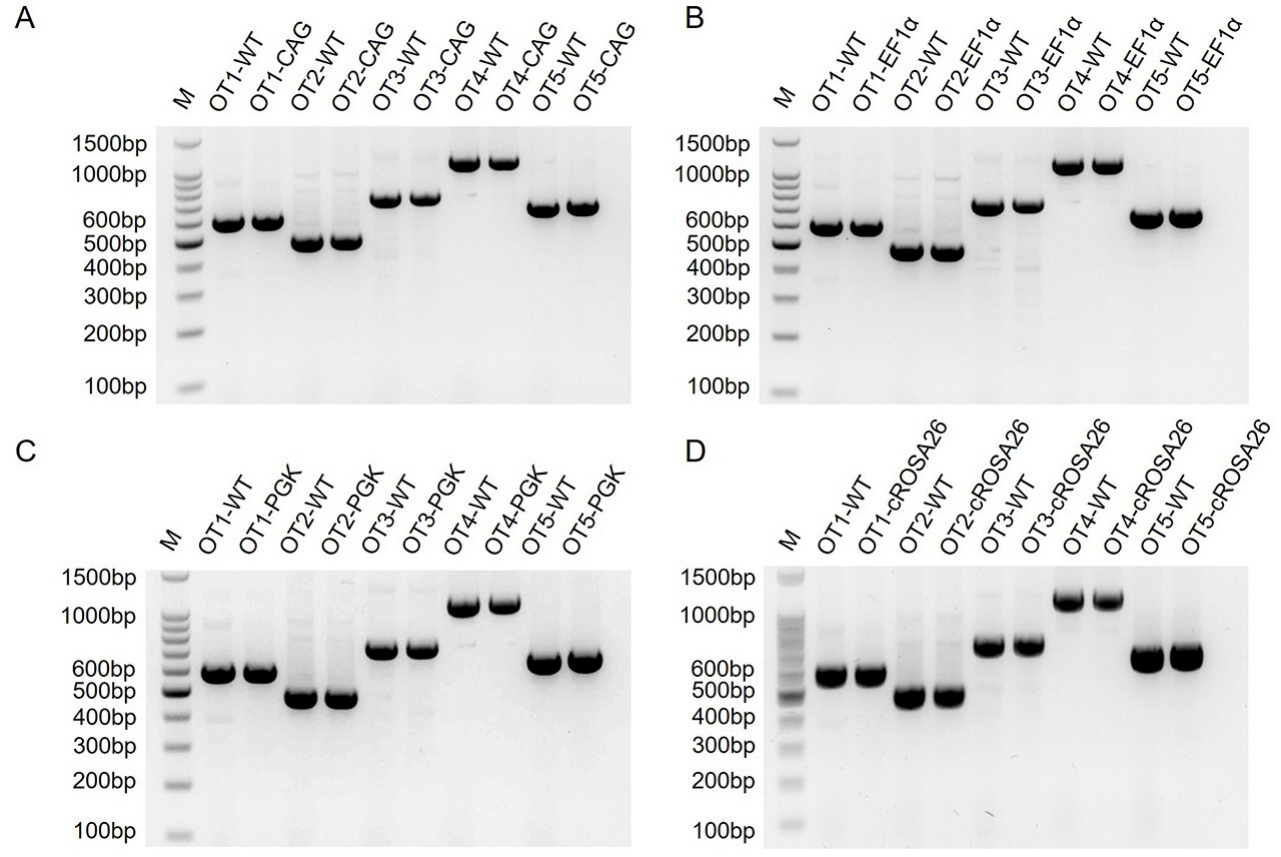
Off-target effect analysis of targeted site-specific integration cell clones. (A) T7E1 assays for the five-potential off-target sites of the correctly CAG-EGFP targeted cells. OT1-OT5 represents 5 potential off-target sites. (B) T7E1 assays for the five-potential off-target sites of the correctly EF1a-EGFP targeted cells. OT1-OT5 represents 5 potential off-target sites. (C) T7E1 assays for the five-potential off-target sites of the correctly PGK-EGFP targeted cells. OT1-OT5 represents 5 potential off-target sites. (D) T7E1 assays for the five-potential off-target sites of the correctly cRosa26-EGFP targeted cells. OT1-OT5 represents 5 potential off-target sites.

Then, 5 potential off-target sites were selected according to an online design tool Cas-OFFinder (http://www.rgenome.net/cas-offinder/), and 3 EGFP-positive cell clones were evaluated. T7E1 assay showed that no off-target mutations were detectable in any of the potential off-target sites or these EGFP-positive cell clones (Fig 3A–3C). The sequences of the top 5 possible off-target sites are listed in Table 3.

**Table 3:** List of the top 5 potential off-target effects of *cROSA26*.

Off-target sites	Sequence	Mismatch
Target sgRNA	GGTGGAGTGAAATGAAGTCCAGG	
Site1	GGTGGAGTGAgAgGAAGTCCTGG	2bp
Site2	GGaGGAtTGAAATGAAGcCCAGG	3bp
Site3	GGTGGtaTGtAATGAAGTCCAGG	3bp
Site4	GaTGGAaTGAAATGAAGgCCAGG	3bp
Site5	GGTaGAGTGAtAaGAAGTCCAGG	3bp

### Characterization of EGFP expressions under the ubiquitous promoter on the cRosa26 locus

**Fig 4:**
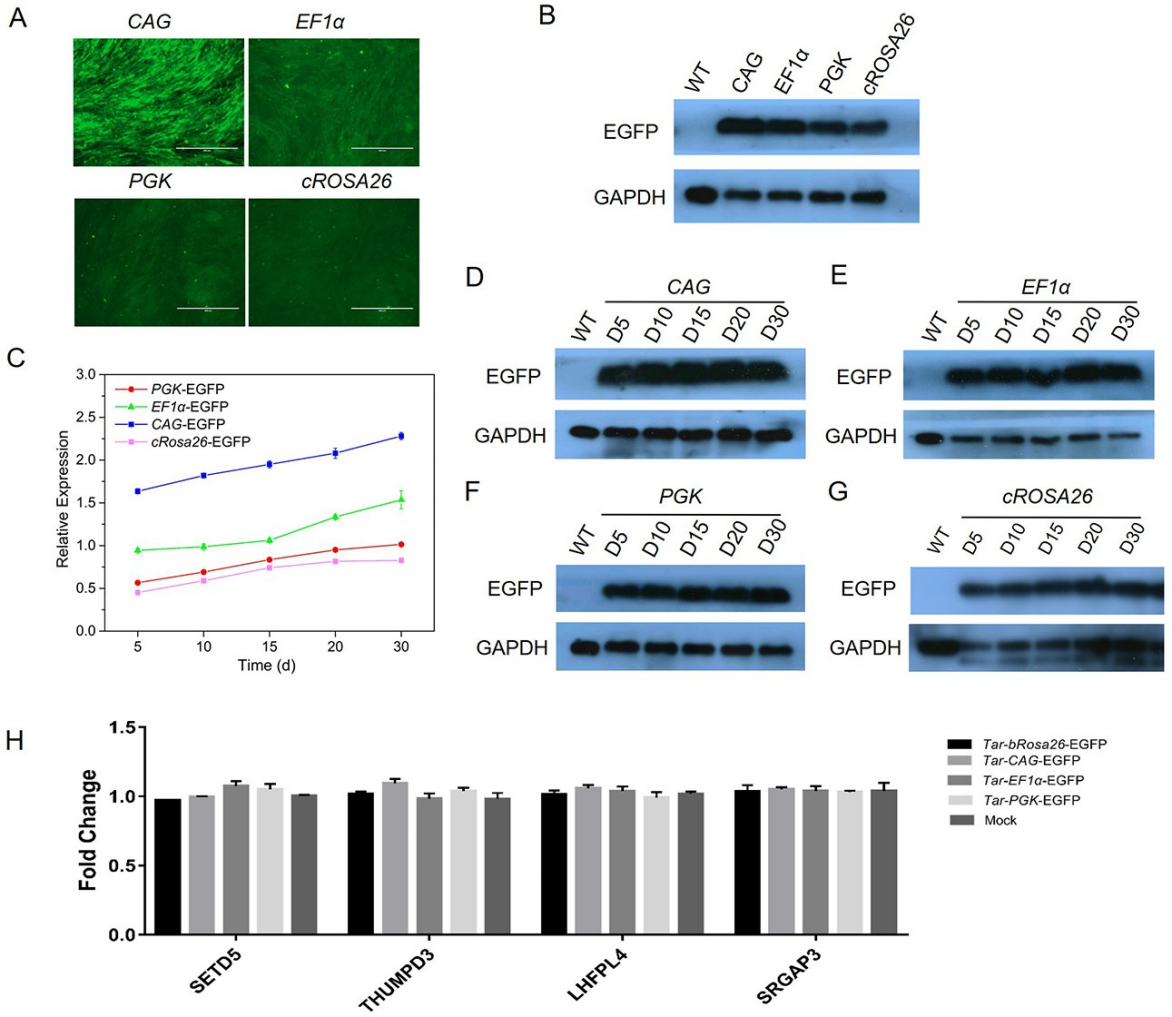
Characterization of EGFP expressions driven by different promoters. (A) EGFP expression in correctly targeted cells 15 d after transduction. The scale bar is 400 μm. (B) EGFP expression in correctly targeted cells detected by Western blot. (C) Stable EGFP expression in correctly targeted cells during 30 d after transduction analyzed by Q-PCR (Mean ± SD, n = 3). (D) Stable EGFP expression in correctly CAG-EGFP targeted cells during 30 d after transduction analyzed by Western blot (Mean ± SD, n = 3). (E) Stable EGFP expression in correctly EF1a-EGFP targeted cells during 30 d after transduction analyzed by Western blot (Mean ± SD, n = 3). (F) Stable EGFP expression in correctly PGK-EGFP targeted cells during 30 d after transduction analyzed by Western blot (Mean ± SD, n = 3). (G) Stable EGFP expression in correctly cRosa26-EGFP targeted cells during 30 d after transduction analyzed by Western blot (Mean ± SD, n = 3). (H) Expression fold change of nearby genes before and after exogenous DNA integration. For all genes, n = 3, mean ± SD, and *p < 0.05. Expression of each gene before integration is used as a control.

**Fig 5:**
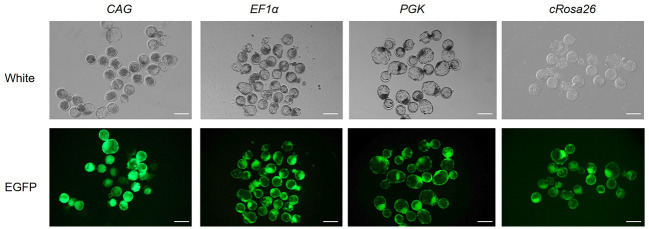
EGFP expressions driven by different promoters in bovine embryos.

Then, EGFP expression with four different promoters (*CMV*, *PGK*, *EF1α* and *cRosa26* promoter) was detected. Fluorescence microscope observation and Western blot analysis showed EGFP expressions were incomparable levels among the three cell lines, and the *CAG* promoter has the highest activity to activate the expression of EGFP in this locus, when compared with the *EF1a*, *PGK* and the *cRosa26* endogenous promoter (Fig 4A and 4B). We further monitored EGFP expression driven by three promoters over a 30-day period. Results demonstrated that all the three promoters could support stable EGFP expression, and exhibited a similar expression trend that increased first and then stabilized (Fig 4C–4G). Subsequently, we used the positive targeted cells as donors to perform NT. As shown in Fig 5, EGFP was expressed in the blastocyst stage. These data suggest that *cRosa26* is suitable for transgene expression driven by different promoters in a high and stable manner.

## Discussion

Continuous and stable expression of foreign genes in vivo or in vitro is important for basic research and application of transgenic technologies. The traditional transgenic technique generally integrates foreign genes into genome by using plasmid transfection, viral transduction or transposons. Among viral transduction, lentivirus-mediated gene transfer has successfully been applied to transgenic cattle [25]. Although viral gene delivery has advantages for efficient genome integration, viral infection may cause activation of proto-oncogene, resulting in potential of tumorigenesis [26]. By zygotic injection using DNA transposons such as the Piggybac (PB), Sleeping beauty (SB), transgenic cattle and multi-gene transgenic cattle could be efficiently generated, which greatly improved the transposon system and broadened the application of zygotic injection to prepare transgenic cattle [27, 28]. However, like the viral transfection, the integration of foreign genes mediated by transposons is still a random integration. The integration site and the copy number can not be accurately controlled, thus leading to the unstable or differentially expression of the exogenous genes. In addition, transgenic individuals prepared by zygotic injection cannot avoid the occurrence of chimerism. Therefore, it is not suitable for the preparation of cattle with long gestation (9 months) and long generation interval (22–26 months).

In recent years, with the rapid development of site-specific integration technology based on homologous recombination technique, it is possible to integrate exogenous genes into a pre-selected locus with a single copy so as to overcome the defects of random integration. Therefore, find a safe locus suitable for insertion and efficient expression of exogenous genes is of great importance. *Rosa26* is the most widely used "safe locus" in mammalian genome. In previous study, the cattle *Rosa26* was proved to be a "safe locus" to support the stable expression of foreign genes under its endogenous promoter [22]. As the expression of foreign genes is often related to promoter activity, therefore, to further improve the expression of foreign genes, a stronger promoter often needed. Meanwhile, exogenous promoters in different species or different locus have different effects on gene expression due to different chromosomal environment, and the insertion of promoters with different intensities at the same site has different effects on the expression of neighboring genes [6, 10, 29]. So, exploring the related characteristics of different active promoters at *cRosa26* locus is necessary and helpful to broaden the wide application of *cRosa26* locus.

The study of mouse *Rosa26* found that the endogenous promoter of *Rosa26* was a medium-strength promoter, and its activity in initiating exogenous gene expression was different from that of the strong promoter CAG [30]. Studies on *Rosa26* in pigs found that the endogenous promoter of *Rosa26* had weaker activity in initiating exogenous gene expression than *EF1a*, *CMV* and *CAG* [31]. For the expression of exogenous genes, we often need to select appropriate promoters. For example, for the expression of exogenous genes in milk, we often need to use mammary gland specific promoters. Therefore, whether *cRosa26* locus can support various promoter to initiate exogenous gene friendly expression need to be studied.

In this study, three broad-spectrum promoters *EF1a*, *CAG* and *PGK* commonly used in large animal transgenic were selected as promoters to drive EGFP gene, as the *cRosa26* endogenous promoter was a control. We successfully constructed four expression units, and targeted to *cRosa26* locus under the mediation of CRISPR/Cas9. According to our statistical results, there was no significant difference in the site-specific integration efficiency of *cRosa26* for the three expression units, and the efficiency was ranged from 78% to 87.5%, which proved that *cRosa26* was a locus that could integrate foreign genes efficiently. EGFP expression analysis was performed on the correctly targeted cells of the four expression units. It was found that the three broad-spectrum promoters could continuously and stably initiate EGFP expression at the *cRosa26* locus, and the activity of *CAG* promoter was higher than that of *EF1a* and *PGK* promoters and even the *cRosa26* endogenous promoter which provided strong evidence that *cRosa26* supported the friendly expression of different expression units. Our findings described here will be useful for a variety of studies using cattle.

## Conclusions

In conclusions, we proved that *cRosa26* was a locus that could integrate different expression units efficiently, and supported the friendly expression of different expression units. Our findings described here will be useful for a variety of studies using cattle.

## Supporting information

S1 Fig CRISPR/Cas9-mediated targeted site-specific integration at the cRosa26 locus. (A) Schematic diagram of the *cRosa26* HDR template vector and strategy for insertion of EGFP expression cassette into the *cRosa26* locus. EGFP, enhanced green fluorescent protein gene; NEO, neomycin-resistance gene. (B) PCR analysis of knock-in cell lines using the primer sets shown in A. M, 1 kb DNA ladder; R1-R4, the positive targeted integration of EGFP-NEO cassettes cell clones; WT, wild-type cFFs; P, the donor vector; H2O was the negative control.
(TIF)

S1 File The whole nucleotide sequence of the *Rosa26*-PGK-EGFP vector. The sequence underlined is the sequence of the 5’ homologous arm targeting the cattle *Rosa26*. The sequence colored in pink indicates the *PGK* Promoter. The sequence colored in green indicates the *EGFP* gene. The sequence colored in yellow indicates the *PGK-NEO-polyA* casstte used for cell selection. The sequence underlined and bolded is the 3’ homologous arm targeting the cattle *Rosa26*.
(PDF)

S2 File The whole nucleotide sequence of the *Rosa26*-CAG-EGFP vector. The sequence underlined is the sequence of the 5’ homologous arm targeting the cattle *Rosa26*. The sequence colored in pink indicates the *CAG* Promoter. The sequence colored in green indicates the *EGFP* gene. The sequence colored in yellow indicates the *PGK-NEO-polyA* casstte used for cell selection. The sequence underlined and bolded is the 3’ homologous arm targeting the cattle *Rosa26*.
(PDF)

S3 File The whole nucleotide sequence of the *Rosa26*-EF1a-EGFP vector. The sequence underlined is the sequence of the 5’ homologous arm targeting the cattle *Rosa26*. The sequence colored in pink indicates the *EF1a* Promoter. The sequence colored in green indicates the *EGFP* gene. The sequence colored in yellow indicates the *PGK-NEO-polyA* casstte used for cell selection. The sequence underlined and bolded is the 3’ homologous arm targeting the cattle *Rosa26*.
(PDF)

S4 File Raw images. (PDF)

S1 Raw images (PDF)
