# Supplementary figures and images for: CRISPR/Cas9-mediated knock-in strategy at the Rosa26 locus in cattle fetal fibroblasts
Source: PLoS One. 2022 Nov 28;17(11):e0276811. doi: 10.1371/journal.pone.0276811 (PMC9704577; doi:10.1371/journal.pone.0276811)

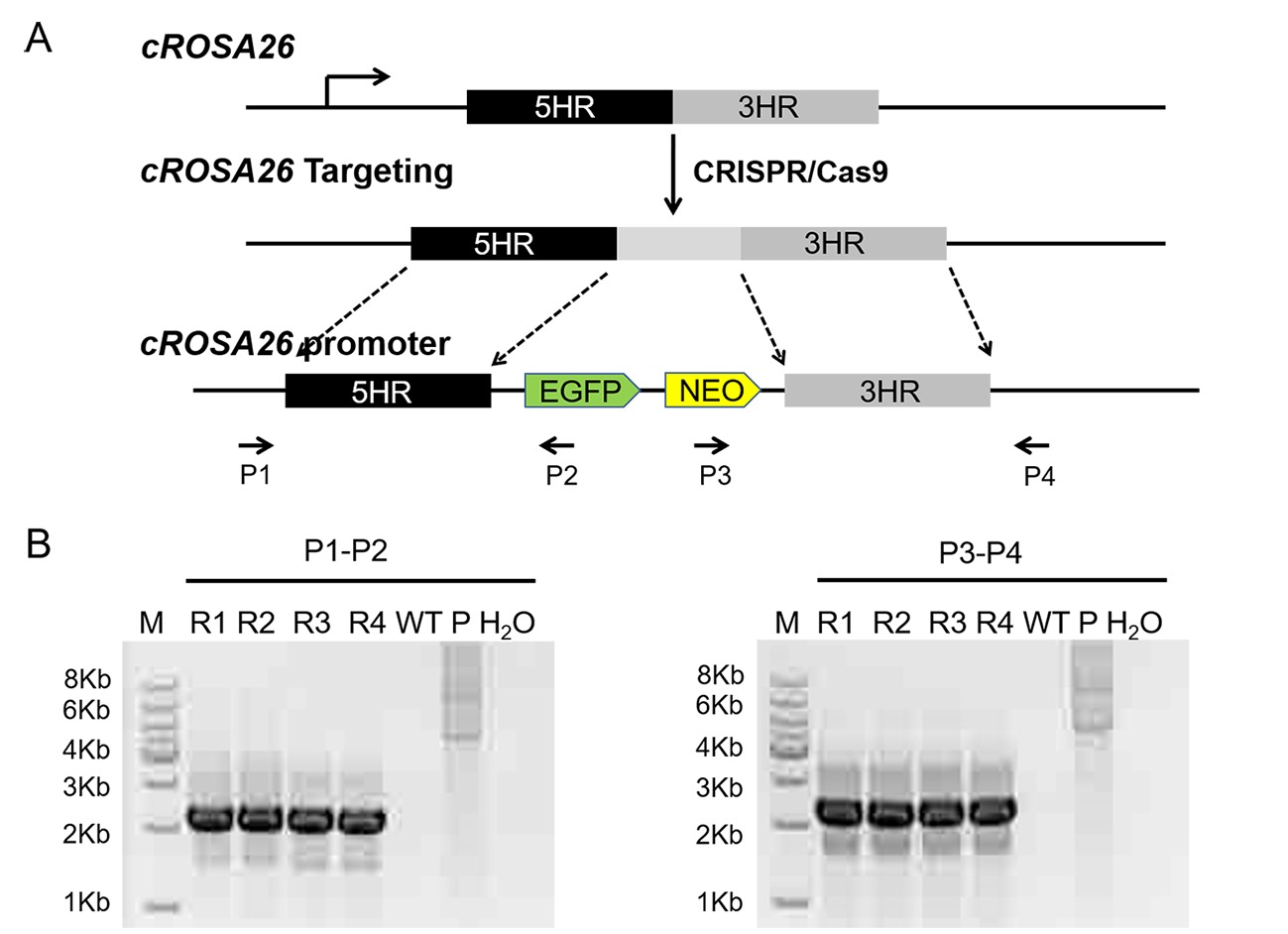

Supplement: S1 Fig — (A) Schematic diagram of the cRosa26 HDR template vector and strategy for insertion of EGFP expression cassette into the cRosa26 locus. EGFP, enhanced green fluorescent protein gene; NEO, neomycin-resistance gene. (B) PCR analysis of knock-in cell lines using the primer sets shown in A. M, 1 kb DNA ladder; R1-R4, the positive targeted integration of EGFP-NEO cassettes cell clones; WT, wild-type cFFs; P, the donor vector; H2O was the negative control. (TIF) [file pone.0276811.s001.tif]
