# Supplementary material for: CRISPR/Cas9-mediated knock-in strategy at the Rosa26 locus in cattle fetal fibroblasts
Source: PLoS One. 2022 Nov 28;17(11):e0276811. doi: 10.1371/journal.pone.0276811 (PMC9704577; doi:10.1371/journal.pone.0276811)
Supplement: S1 File — The sequence underlined is the sequence of the 5’ homologous arm targeting the cattle Rosa26. The sequence colored in pink indicates the PGK Promoter. The sequence colored in green indicates the EGFP gene. The sequence colored in yellow indicates the PGK-NEO-polyA casstte used for cell selection. The sequence underlined and bolded is the 3’ homologous arm targeting the cattle Rosa26. (PDF) [file pone.0276811.s002.pdf]

ctgacgcgccctgtagcggcgcattaagcgcggcgggtgtggtggttacgcgcagcgtgaccgctacacttgccagcgccct
agcgcccgctcctttcgctttcttcccttcctttctcgccacgttcgccggctttccccgtcaagctctaaatcgggggctccctttagg
gttccgatttagtgctttacggcacctcgaccccaaaaaacttgattagggtgatggttcacgtagtgggccatcgccctgataga
cggtttttcgccctttgacgttggagtccacgttctttaatagtggactcttgttccaaactggaacaacactcaaccctatctcggtc
tattcttttgatttataagggattttgccgatttcggcctattggttaaaaaatgagctgatttaacaaaaatttaacgcgaattttaac
aaaatattaacgcttacaatttccattcgccattcaggctgcgcaactgttgggaagggcgatcggtgcgggcctcttcgctatta
cgccagctggcgaaagggggatgtgctgcaaggcgattaagttgggtaacgccagggttttcccagtcacgacgttgtaaaa
cgacggccagtgagcgcgcgtaatacgactcactatagggcgaattggagctccaccgcggGGCGCGccggtaggggaa
gcggaactctGGTGGGAGGGGGTCCGGCGGATTGGTGGGGGGATGGGTGGCTGAGGTCGTCTGG
CCGGTACCTGGGGGTCGCTTTCCCCGGTGGGAAGAGGGGAGAATAGCGTTTGTTACGCTGAAA
GGGAGAGAGGTGGTCAGAGGCAGGCGGGAGTGCGGCCCGCCTTGCGGCAGCCGAAGGGGGA
GGGAGAAGGGAGCGGAAAAGGCTCGAATCCGGACGGAGCCATTGCTCCTGCAGAGGGAGGA
GCGCTTCCGGCTCTTATCTTGTCACTGATTGGTTGCTGCTGCTCCCGCCGTGTGTGAAAACACAA
ATGGCGTGTTTTGGTTGGAGTAAAGCGCCTGTCAGTTACAGCCTCGGGAGTGCGCAGCCGCCTA
GGGACTCTCGCATTGCCCACTGGGTGGGTGCTTAGGTAGGTAGGGTGGAGAGAGACTTGGATG
AGCAGGCGCGGTCGGCCTCCCACGGGGAGGTAGGGCTGGGGGTGGGAAGGGAGGGTCAGTG
AAAGTGGCTTCGCGCGGGCGTCCTACCACCCACCCCTTCCTTCGGGGGAGTCGGTTTACCCGCC
GCCTGCTTGGCTTCGGCATCTGATTGGCTGCTGAAGCTCAGGGAACGGCCCCTTGTTATTGGCT
CGGGTCCCAAATGAGCGAAACCACTGCGCGGGTCGGCGGGGAGGCGGTCGTTGGTACGATCC
TCCCCGAGACCCAGCGCCGCAGTGTCTGGCCCCGCGCCCCTGCGCAACGTGGCAGGAAGCGC
GCGCTGGAGGTGGGGGCGGACTGCCGGGCCGAGGATTCTGGGTGGTGGCGATTGCGGCTCCG
CCCTGGGCGCCCGCTGCCTGAAGGACAAGACTAGCCCGACCTGCTCCTGGACCCGTGGGGCTG
AAGGGAGGAGTGGGGGTCGGTGCCGCTGGCTTGTGGGTGGGAGGTGCATGTTCTCCAAAAATC
GGCGCGAGCTGCAATCCTGAGGTGGCTGCAGTGGAGGAGGCGGAGAGAAGGCCGCACCCTTC
TCAGCAGGGGGAGGGGAGTGCCGCAATACCTTTATGGGAGTTCTCTGCTGCCTCCTGTCTCCTA
AGGACCGCCCTGGGCCTAGAAGAATCCCTCCCTCCCCCGCGATCTCGTCATCGCCTCCATGTCG
AGTCTCGATTATGGGCGGGATTCTTTTGCCCAGGCTTAACCTTATTCCTGGGCGTTGTCCTGCAG
GGGATCGAGCAGGTATAAGACTTAGAGGACGAACCCAATTTCTTTTATCTTCCCACAGGCTTGA
GTTTGTGTCACAAAATAATTATAATGGggtggtggagtgaaatgaagtggCGCGCCATTAATGGTACCA
ATTCTACCGGGTAGGGGAGGcgcttttcccaaggcagtctggagcatgcgctttagcagccccgctggcacttggcgc
tacacaagtggcctctggcctcgcacacattccacatccaccggtagcgccaaccggctccgttctttggtggccccttcgcgcca
ccttctactcctcccctagtcaggaagttcccccccgccccgcagctcgcgtcgtgcaggacgtgacaaatggaagtagcacgtc
tcactagtctcgtgcagatggacagcaccgctgagcaatggaagcgggtaggcctttggggcagcggccaatagcagctttg
ctccttcgctttctgggctcagcagctgggaagggtgggtccgggggcgggctcaggggcgggctcaggggcggggcgg
gcgcccgaaggtcctccggaggcccggcattctgcacgcttcaaaagcgcacgtctgccgcgctgttctcctcttcctcATCTC
CGGGCCTTTCGACCTGAATTCACCGGTCGCCACCATGGTGAGCAAGGGCGAGGAGCTGTTCAC
CGGGGTGGTGCCCATCCTGGTCGAGCTGGACGGCGACGTAAACGGCCACAAGTTCAGCGTGTC
CGGCGAGGGCGAGGGCGATGCCACCTACGGCAAGCTGACCCTGAAGTTCATCTGCACCACCG
GCAAGCTGCCCGTGCCCTGGCCCACCCTCGTGACCACCCTGACCTACGGCGTGCAGTGCTTCA
GCCGCTACCCCGACCACATGAAGCAGCACGACTTCTTCAAGTCCGCCATGCCCGAAGGCTACG
TCCAGGAGCGCACCATCTTCTTCAAGGACGACGGCAACTACAAGACCCGCGCCGAGGTGAAGT
TCGAGGGCGACACCCTGGTGAACCGCATCGAGCTGAAGGGCATCGACTTCAAGGAGGACGGC
AACATCCTGGGGCACAAGCTGGAGTACAACTACAACAGCCACAACGTCTATATCATGGCCGAC
AAGCAGAAGAACGGCATCAAGGTGAACTTCAAGATCCGCCACAACATCGAGGACGGCAGCGT

GCAGCTCGCCGACCACTACCAGCAGAACACCCCCATCGGCGACGGCCCCGTGCTGCTGCCCGA
CAACCACTACCTGAGCACCCAGTCCGCCCTGAGCAAAGACCCCAACGAGAAGCGCGATCACAT
GGTCCTGCTGGAGTTCGTGACCGCCGCCGGGATCACTCTCGGCATGGACGAGCTGTACAAGTA
AAGCGGCCGCGACTCTAGATCATAATCAGCCATACCACATTTGTAGAGGTTTTACTTGCTTTAAA
AAACCTCCCACACCTCCCCCTGAACCTGAAACATAAAATGAATGCAATTGTTGTTGTTAACTTGT
TTATTGCAGCTTATAATGGTTACAAATAAAGCAATAGCATCACAAATTTCACAAATAAAGCATTT
TTTTCACTGCATTCTAGTTGTGGTTTGTCCAAACTCATCAATGTATCTTAAGGCggccgctctagaacta
gtggatccggaacccttaatataacttcgtataatgtatgctatacgaagttattaggtccctcgacctgcaggaattctaccgggt
aggggaggcgcttttcccaaggcagtctggagcatgcgctttagcagccccgctgggcacttggcgctacacaagtggcctct
ggcctcgcacacattccacatccaccggtaggcgccaaccggctccgttctttggtggccccttcgcgccaccttctactcctccc
tagtcaggaagttcccccccgccccgcagctcgcgtcgtgcaggacgtgacaaatggaagtagcacgtctcactagtctcgtgc
agatggacagcaccgctgagcaatggaagcgggtaggcctttggggcagcggccaatagcagctttgctccttcgctttctgg
gctcagaggctgggaaggggtgggtccgggggcgggctcaggggcgggctcaggggcggggcgggcgcccgaaggt
cctccggaggcccggcattctgcacgcttcaaaagcgcacgtctgccgcgctgttctcctcttcctcatctccgggcctttcgacct
gcagccaatatgggatcggccattgaacaagatggattgcacgcaggttctccggccgcttgggtggagaggctattcggcta
tgactgggcacaacagacaatcggctgctctgatgccgccgtgttccggctgtcagcgcaggggcgcccggttctttttgtcaa
gaccgacctgtccggtgccctgaatgaactgcaggacgaggcagcgcggctatcgtggctggccacgacgggcgttccttgc
gcagctgtgctcgacgttgtcactgaagcgggaagggactggctgctattgggcgaagtgccggggcaggatctcctgtcat
ctcaccttgctcctgccgagaaagtatccatcatggctgatgcaatgcggcggctgcatacgcttgatccggctacctgcccattc
gaccaccaagcgaaacatcgcatcgagcgagcacgtactcggatggaagccggtcttgtcgatcaggatgatctggacgaag
agcatcaggggctcgcgccagccgaactgttcgccaggctcaaggcgcgcatgcccgacggcgatgatctcgtcgtgaccca
tggcgatgcctgcttgccgaatatcatggtggaaaatggccgcttttctggattcatcgactgtggccggctgggtgtggcgga
ccgctatcaggacatagcgttggctacccgtgatattgctgaagagcttggcggcgaatgggctgaccgcttcctcgtgctttac
ggtatcgccgctcccgattcgcagcgcatcgccttctatcgccttcttgacgagttcttctgaggggatcaattctctagagctcgct
gatcagcctcgactgtgccttctagttgccagccatctgttgtttgcccctcccccgtgccttccttgaccctggaaggtgccactcc
cactgtcctttcctaataaaatgaggaaattgcatcgcattgtctgagtaggtgtcattctattctggggggtggggtggggcag
gacagcaagggggaggattgggaagacaatagcaggcatgctggggatgcggtgggctctatggcttctgaggcggaaa
gaaccagctggggctcgaatcaagctgatccggaacccttaatataacttcgtataatgtatgctatacgaagttattaggtccctc
gacctgcagcccaagctagc**ccaggcaacacctaggacttaTTTTATGCAGCGAGACTGCGAGTTACTACTT**
CTTAACATCCTTTTGTTGCATATTTTCCAGGAGATTGAGAGAGAGGTTAAAAGCTTGATCTCC
TGAATTTTATACTCTCCCCATTTGAGACAGTTGAGAAATAGGTTAAAGGCATGCTCTCTTGA
GTTCCCCATTTGAGACCGTTGCTACACCGCCAAAACAGAGCATTTTAGATTAGATCTTAAAA
ATTTAATTCCCCACCCTTGCATTCTCAGAGTCAGGCCTTTTAGCAACTCTCACTCATACTTTCA
GCCCATTTTCTGTTTGTTACACTTGCTCATCTTGTCCCAGTCATACCATTGGCTTTCTCCTCTCC
TGTTTTTGGTATCCCGGTGAGTCATGAAACCAGACAGGTTTCACCACCAATTAAGGCTACCC
AGCTCGAGCATAGGCTTCACTCTTGCCCAGAAATGCATTTATTCCTCTTTTATGGATATTCTG
GAGTCTTTACCTTGATTTTCATTTAATTTTTTAACCTCAGCTGGGATTCTACTGACCCTCTTAA
TAGTCCAGATGATCTTGACGACTGCTTTGCTGAGAACCGGACGTGAGGTTCAGCAACATCTC
TTTTATATCCTTAGAATACCTTTCAACCCATTTTCATTGATATGCTTATGAGTTAGTAATCAAG
CTCAGTTGCCATAAGGCTAGTATCCTTCGAACTAGGATCTCTTGTCTCTGGTATCTGCTGATA
CAACTTTCATATGTGTCCAGGACAGTAGTTCTCATACAAAGATAACAGCATGGAAGTAACCG
ATCCAACTCCTTTACTGCCTGGTAACTACTGACAGGATGCGTTCCATCATCACAAATGTGAT
GTACAAGGTCCCTCAATGGACTAACCTCACCTTAACAGCCTTTTTGTTTGTGACAGTTTTCCA
CATACACACCCAAACAATATTATTGGACCTCTTTGTAGGGGTGGTTCCTCCTGGAGTGCTAC

CCTTGATAGTCCTTACCCTTCCAATAAAGACTGTTAAAACTCAAATATCATCTCCCCTATGAT CTTGCCTTCTGTGGTCTATGCTTTAAGCTAGAATCCCCTTTCTCTTGGTCCCATACATAGCAG GTTGAATCATAGCACTTCTCAGGTGGTTGTCAGTGCTTATTTAAATTATCTTAGCTATTCTGA GCTGCTTGTTGAGTGTTGTACCTAAGTTCCTAgtgtatttcttgatggctagcgctagcttatcgataccgtcg acggtatcgataagcttgatatcgaattctaccgggtaggggaggcgcttttcccaaggcagtctggagcatgcgctttagcag ccccgctgggcacttggcgctacacaagtggcctctggcctcgcacacattccacatccaccggtaggcgccaaccggctccgt tctttggtggccccttcgcgccaccttctactcctcccctagtcaggaagttcccccccgccccgcagctcgcgtcgtgcaggacgt gacaaatggaagtagcacgtctcactagtctcgtgcagatggacagcaccgctgagcaatggaagcgggtaggcctttgggg cagcggccaatagcagctttgctccttcgctttctgggctcagaggctgggaaggggtgggtccgggggcgggctcagggg cgggctcaggggcggggcgggcgcccgaaggtcctccggaggcccggcattctgcacgcttcaaaagcgcacgtctgccg cgctgttctcctcttcctcatctccgggcctttcgacctgcaggtcctcgccatggatcctgatgatgttgttgattcttctaaatctttt gtgatggaaaacttttcttcgtaccacgggactaaacctggttatgtagattccattcaaaaaggtatacaaaagccaaaatctgg tacacaaggaaattatgacgatgattggaaagggttttatagtaccgacaataaatacgacgctgcgggatactctgtagataat gaaaacccgctctctggaaaagctggaggcgtggtcaaagtgacgtatccaggactgacgaaggttctcgcactaaaagtgg ataatgccgaaactattaagaaagagttaggtttaagtctcactgaaccgttgatggagcaagtcggaacggaagagtttatca aaaggttcggtgatggtgcttcgcgtgtagtgctcagccttcccttcgctgaggggagttctagcgttgaatatattaataactgg gaacaggcgaaagcgttaagcgtagaacttgagattaattttgaaacccgtggaaaacgtggccaagatgcgatgtatgagta tatggctcaagcctgtgcaggaaatcgtgtcaggcgatctctttgtgaaggaaccttacttctgtggtgtgacataattggacaaa ctacctacagagatttaaagctctaaggtaaatataaaatttttaagtgtataatgtgttaaactactgattctaattgtttgtgtatttt agattccaacctatggaactgatgaatgggagcagtggtggaatgcagatcctagagctcgctgatcagcctcgactgtgcctt ctagttgccagccatctgttgtttgcccctcccccgtgccttccttgaccctggaaggtgccactcccactgtcctttcctaataaaat gaggaaattgcatcgcattgtctgagtaggtgtcattctattctggggggtggggtggggcaggacagcaagggggaggatt gggaagacaatagcaggcatgctggggatgcggtgggctctatggcttctgaggcggaaagaaccagctggggctcgagg gggggcccggtacccagcttttgttccctttagtgagggttaattgcgcgcttggcgtaatcatggtcatagctgtttcctgtgtga aattgttatccgctcacaattccacacaacatacgagccggaagcataaagtgtaaagcctggggtgcctaatgagtgagctaa ctcacattaattgcgttgcgctcactgcccgctttccagtcgggaaacctgtcgtgccagctgcattaatgaatcggccaacgcgc ggggagaggcggtttgcgtattgggcgctcttccgcttcctcgctcactgactcgctgcgctcggtcgttcggctgcggcgagc ggtatcagctcactcaaaggcggtaatacggttatccacagaatcaggggataacgcaggaaagaacatgtgagcaaaagg ccagcaaaaggccaggaaccgtaaaaaggccgcgttgctggcgtttttccataggctccgcccccctgacgagcatcacaaaa atcgacgctcaagtcagaggtggcgaaacccgacaggactataaagataccaggcgtttccccctggaagctccctcgtgcgc tctcctgttccgaccctgccgcttaccggatacctgtccgcctttctcccttcgggaagcgtggcgctttctcatagctcacgctgta ggtatctcagttcggtgtaggtcgttcgctccaagctgggctgtgtgcacgaaccccccgttcagcccgaccgctgcgccttatcc ggtaactatcgtcttgagtccaacccggtaagacacgacttatcgccactggcagcagccactggtaacaggattagcagagc gaggtatgtaggcggtgctacagagttcttgaagtggtggcctaactacggctacactagaagaacagtatttggtatctgcgct ctgctgaagccagttaccttcggaaaaagagttggtagctcttgatccggcaaacaaaccaccgctggtagcggtggtttttttgt ttgcaagcagcagattacgcgcagaaaaaaaggatctcaagaagatcctttgatcttttctacggggtctgacgctcagtggaa cgaaaactcacgttaagggattttggtcatgagattatcaaaaaggatcttcacctagatccttttaaattaaaaatgaagttttaaa tcaatctaaagtatatatgagtaaacttggtctgacagttaccaatgcttaatcagtgaggcacctatctcagcgatctgtctatttc gttcatccatagttgcctgactccccgtcgtgtagataactacgatacgggagggcttaccatctggccccagtgctgcaatgata ccgcgagacccacgctcaccggctccagatttatcagcaataaaccagccagccggaagggccgagcgcagaagtggtcct gcaactttatccgcctccatccagtctattaattgttgccgggaagctagagtaagtagttcgccagttaatagtttgcgcaacgtt gttgccattgctacaggcatcgtggtgtcacgctcgtcgtttggtatggcttcattcagctccggttcccaacgatcaaggcgagtt acatgatcccccatgttgtgcaaaaaagcggttagctccttcggtcctccgatcgttgtcagaagtaagttggccgcagtgttatc actcatggttatggcagcactgcataattctcttactgtcatgccatccgtaagatgcttttctgtgactggtgagtactcaaccaag

tcattctgagaatagtgtatgcggcgaccgagttgctcttgcccggcgtcaatacgggataataccgcgccacatagcagaactt
taaaagtgctcatcattggaaaacgttcttcggggcgaaaactctcaaggatcttaccgctgttgagatccagttcgatgtaaccc
actcgtgcacccaactgatcttcagcatcttttactttcaccagcgtttctgggtgagcaaaaacaggaaggcaaaatgccgcaa
aaaagggaataagggcgacacggaaatgttgaatactcatactcttcctttttcaatattattgaagcatttatcagggttattgtct
catgagcggatacatatttgaatgtatttagaaaaataaacaaataggggttccgcgcacatttccccgaaaagtgccac
