# Supplementary material for: CRISPR/Cas9-mediated knock-in strategy at the Rosa26 locus in cattle fetal fibroblasts
Source: PLoS One. 2022 Nov 28;17(11):e0276811. doi: 10.1371/journal.pone.0276811 (PMC9704577; doi:10.1371/journal.pone.0276811)
Supplement: S2 File — The sequence underlined is the sequence of the 5’ homologous arm targeting the cattle Rosa26. The sequence colored in pink indicates the CAG Promoter. The sequence colored in green indicates the EGFP gene. The sequence colored in yellow indicates the PGK-NEO-polyA casstte used for cell selection. The sequence underlined and bolded is the 3’ homologous arm targeting the cattle Rosa26. (PDF) [file pone.0276811.s003.pdf]

ctgacgcgccctgtagcggcgcattaagcgcggcgggtgtggtggttacgcgcagcgtgaccgctacacttgccagcgccct
agcgcccgctcctttcgctttcttcccttcctttctcgccacgttcgccggctttccccgtcaagctctaaatcgggggctccctttagg
gttccgatttagtgctttacggcacctcgaccccaaaaaacttgattagggtgatggttcacgtagtgggccatcgccctgataga
cggtttttcgccctttgacgttggagtccacgttctttaatagtggactcttgttccaaactggaacaacactcaaccctatctcggtc
tattcttttgatttataagggattttgccgatttcggcctattggttaaaaaatgagctgatttaacaaaaatttaacgcgaattttaac
aaaatattaacgcttacaatttccattcgccattcaggctgcgcaactgttgggaagggcgatcggtgcgggcctcttcgctatta
cgccagctggcgaaagggggatgtgctgcaaggcgattaagttgggtaacgccagggttttcccagtcacgacgttgtaaaa
cgacggccagtgagcgcgcgtaatacgactcactatagggcgaattggagctccaccgcggGGCGCGccggtaggggga
gcggaactctGGTGGGAGGGGGTCCGGCGGATTGGTGGGGGGATGGGTGGCTGAGGTCGTCTGG
CCGGTACCTGGGGGTCGCTTTCCCCGGTGGGAAGAGGGGAGAATAGCGTTTGTTACGCTGAAA
GGGAGAGAGGTGGTCAGAGGCAGGCGGGAGTGCGGCCCGCCTTGCGGCAGCCGAAGGGGGA
GGGAGAAGGGAGCGGAAAAGGCTCGAATCCGGACGGAGCCATTGCTCCTGCAGAGGGAGGA
GCGCTTCCGGCTCTTATCTTGTCACTGATTGGTTGCTGCTGCTCCCGCCGTGTGTGAAAACACAA
ATGGCGTGTTTTGGTTGGAGTAAAGCGCCTGTCAGTTACAGCCTCGGGAGTGCGCAGCCGCCTA
GGGACTCTCGCATTGCCCACTGGGTGGGTGCTTAGGTAGGTAGGGTGGAGAGAGACTTGGATG
AGCAGGCGCGGTCGGCCTCCCACGGGGAGGTAGGGCTGGGGGTGGGAAGGGAGGGTCAGTG
AAAGTGGCTTCGCGCGGGCGTCCTACCACCCACCCCTTCCTTCGGGGGAGTCGGTTTACCCGCC
GCCTGCTTGGCTTCGGCATCTGATTGGCTGCTGAAGCTCAGGGAACGGCCCCTTGTTATTGGCT
CGGGTCCCAAATGAGCGAAACCACTGCGCGGGTCGGCGGGGAGGCGGTCGTTGGTACGATCC
TCCCCGAGACCCAGCGCCGCAGTGTCTGGCCCCGCGCCCCTGCGCAACGTGGCAGGAAGCGC
GCGCTGGAGGTGGGGGCGGACTGCCGGGCCGAGGATTCTGGGTGGTGGCGATTGCGGCTCCG
CCCTGGGCGCCCGCTGCCTGAAGGACAAGACTAGCCCGACCTGCTCCTGGACCCGTGGGGCTG
AAGGGAGGAGTGGGGGTCGGTGCCGCTGGCTTGTGGGTGGGAGGTGCATGTTCTCCAAAAATC
GGCGCGAGCTGCAATCCTGAGGTGGCTGCAGTGGAGGAGGCGGAGAGAAGGCCGCACCCTTC
TCAGCAGGGGGAGGGGAGTGCCGCAATACCTTTATGGGAGTTCTCTGCTGCCTCCTGTCTCCTA
AGGACCGCCCTGGGCCTAGAAGAATCCCTCCCTCCCCCGCGATCTCGTCATCGCCTCCATGTCG
AGTCTCGATTATGGGCGGGATTCTTTTGCCCAGGCTTAACCTTATTCCTGGGCGTTGTCCTGCAG
GGGATCGAGCAGGTATAAGACTTAGAGGACGAACCCAATTTCTTTTATCTTCCCACAGGCTTGA
GTTTGTGTCACAAAATAATTATAATGGggtggtggagtgaaatgaagtggCGCGCCCTCGACATTGATT
ATTGACTAGTTATTAATAGTAATCAATTACGGGGTCATTAGTTCATAGCCCATATATGGAGTTCC
GCGTTACATAACTTACGGTAAATGGCCCGCCTGGCTGACCGCCCAACGACCCCCGCCCATTGA
CGTCAATAATGACGTATGTTCCCATAGTAACGCCAATAGGGACTTTCCATTGACGTCAATGGGT
GGACTATTTACGGTAAACTGCCCACTTGGCAGTACATCAAGTGTATCATATGCCAAGTACGCCC
CCTATTGACGTCAATGACGGTAAATGGCCCGCCTGGCATTATGCCCAGTACATGACCTTATGGG
ACTTTCCTACTTGGCAGTACATCTACGTATTAGTCATCGCTATTACCATGGGTCGAGGTGAGCCC
CACGTTCTGCTTCACTCTCCCCATCTCCCCCCCCTCCCCACCCCCAATTTTGTATTTATTTATTTTT
TAATTATTTTGTGCAGCGATGGGGGCGGGGGGGGGGGGGCGCGCGCCAGGCGGGGCGGGG
CGGGGCGAGGGGCGGGGCGGGGCGAGGCGGAGAGGTGCGGCGGCAGCCAATCAGAGCGGC
GCGCTCCGAAAGTTTCCTTTTATGGCGAGGCGGCGGCGGCGGCGGCCCTATAAAAAGCGAAGC
GCGCGGCGGGCGGGAGTCGCTGCGTTGCCTTCGCCCCGTGCCCCGCTCCGCGCCGCCTCGCGC
CGCCCGCCCCGGCTCTGACTGACCGCGTTACTCCCACAGGTGAGCGGGCGGGACGGCCCTTCT
CCTCCGGGCTGTAATTAGCGCTTGGTTTAATGACGGCTCGTTTCTTTTCTGTGGCTGCGTGAAAG
CCTTAAAGGGCTCCGGGAGGGCCCTTTGTGCGGGGGGGAGCGGCTCGGGGGGTGCGTGCGTG
TGTGTGTGCGTGGGGAGCGCCGCGTGCGGCCCGCGCTGCCCGGCGGCTGTGAGCGCTGCGGG

CGCGGCGCGGGGCTTTGTGCGCTCCGCGTGTGCGCGAGGGGAGCGCGGCCGGGGGCGGTGCC
CCGCGGTGCGGGGGGGCTGCGAGGGGAACAAAGGCTGCGTGCGGGGTGTGTGCGTGGGGG
GTGAGCAGGGGGTGTGGGCGCGGCGGTCGGGCTGTAACCCCCCCCTGCACCCCCCTCCCCGA
GTTGCTGAGCACGGCCCGGCTTCGGGTGCGGGGCTCCGTGCGGGGCGTGGCGCGGGGCTCGC
CGTGCCGGGCGGGGGGTGGCGGCAGGTGGGGGTGCCGGGCGGGGCGGGGCCGCCTCGGGCC
GGGGAGGGCTCGGGGGAGGGGCGCGGCGGCCCCGGAGCGCCGGCGGCTGTCGAGGCGCGG
CGAGCCGCAGCCATTGCCTTTTATGGTAATCGTGCGAGAGGGCGCAGGGACTTCCTTTGTCCCA
AATCTGGCGGAGCCGAAATCTGGGAGGCGCCGCCGCACCCCCTCTAGCGGGCGCGGGCGAAG
CGGTGCGGCGCCGGCAGGAAGGAAATGGGCGGGGAGGGCCTTCGTGCGTCGCCGCGCCGCC
GTCCCCTTCTCCATCTCCAGCCTCGGGGCTGCCGCAGGGGGACGGCTGCCTTCGGGGGGGACG
GGGCAGGGCGGGGTTCGGCTTCTGGCGTGTGACCGGCGGCTCTAGAGCCTCTGCTAACCATGT
TCATGCCTTCTTCTTTTTCCTACAGCTCCTGGGCAACGTGCTGGTTATTGTGCTGTCTCATCATTTT
GGCAAAGAATTCACCGGTCGCCACCATGGTGAGCAAGGGCGAGGAGCTGTTCACCGGGGTGG
TGCCCATCCTGGTCGAGCTGGACGGCGACGTAAACGGCCACAAGTTCAGCGTGTCCGGCGAGG
GCGAGGGCGATGCCACCTACGGCAAGCTGACCCTGAAGTTCATCTGCACCACCGGCAAGCTGC
CCGTGCCCTGGCCCACCCTCGTGACCACCCTGACCTACGGCGTGCAGTGCTTCAGCCGCTACCC
CGACCACATGAAGCAGCACGACTTCTTCAAGTCCGCCATGCCCGAAGGCTACGTCCAGGAGCG
CACCATCTTCTTCAAGGACGACGGCAACTACAAGACCCGCGCCGAGGTGAAGTTCGAGGGCGA
CACCCTGGTGAACCGCATCGAGCTGAAGGGCATCGACTTCAAGGAGGACGGCAACATCCTGGG
GCACAAGCTGGAGTACAACTACAACAGCCACAACGTCTATATCATGGCCGACAAGCAGAAGAA
CGGCATCAAGGTGAACTTCAAGATCCGCCACAACATCGAGGACGGCAGCGTGCAGCTCGCCGA
CCACTACCAGCAGAACACCCCCATCGGCGACGGCCCCGTGCTGCTGCCCGACAACCACTACCT
GAGCACCCAGTCCGCCCTGAGCAAAGACCCCAACGAGAAGCGCGATCACATGGTCCTGCTGG
AGTTCGTGACCGCCGCCGGGATCACTCTCGGCATGGACGAGCTGTACAAGTAAAGCGGCCGCG
ACTCTAGATCATAATCAGCCATACCACATTTGTAGAGGTTTTACTTGCTTTAAAAAACCTCCCAC
ACCTCCCCCTGAACCTGAAACATAAAATGAATGCAATTGTTGTTGTTAACTTGTTTATTGCAGCTT
ATAATGGTTACAAATAAAGCAATAGCATCACAAATTTCACAAATAAAGCATTTTTTTCACTGCAT
TCTAGTTGTGGTTTGTCCAAACTCATCAATGTATCTTAAGGCggccgctctagaactagtggatccggaacc
cttaatataacttcgtataatgtatgctatacgaagttattaggtccctcgacctgcaggaattctaccgggtaggggaggcgcttt
tcccaaggcagtctggagcatgcgctttagcagccccgctgggcacttggcgctacacaagtggcctctggcctcgcacacatt
ccacatccaccggtaggcgccaaccggctccgttctttggtggccccttcgcgccaccttctactcctcccctagtcaggaagttcc
cccccgccccgcagctcgcgtcgtgcaggacgtgacaaatggaagtagcacgtctcactagtctcgtgcagatggacagcacc
gctgagcaatggaagcgggtaggcctttggggcagcggccaatagcagctttgctccttcgctttctgggctcagaggctggg
aaggggtgggtccgggggcgggctcaggggcgggctcaggggcggggcgggcgcccgaaggtcctccggaggcccg
gcattctgcacgcttcaaaagcgcacgtctgccgcgctgttctcctcttcctcatctccgggcctttcgacctgcagccaatatggg
atcggccattgaacaagatggattgcacgcaggttctccggccgcttgggtggagaggctattcggctatgactgggcacaac
agacaatcggctgctctgatgccgccgtgttccggctgtcagcgcaggggcgcccggttctttttgtcaagaccgacctgtccg
gtgccctgaatgaactgcaggacgaggcagcgcggctatcgtggctggccacgacgggcgttccttgcgcagctgtgctcga
cgttgtcactgaagcgggaagggactggctgctattgggcgaagtgccggggcaggatctcctgtcatctcaccttgctcctgc
cgagaaagtatccatcatggctgatgcaatgcggcggctgcatacgcttgatccggctacctgcccattcgaccaccaagcgaa
acatcgcatcgagcgagcacgtactcggatggaagccggtcttgtcgatcaggatgatctggacgaagagcatcaggggctc
gcgccagccgaactgttcgccaggctcaaggcgcgcatgcccgacggcgatgatctcgtcgtgacccatggcgatgcctgctt
gccgaatatcatggtggaaaatggccgcttttctggattcatcgactgtggccggctgggtgtggcggaccgctatcaggacat
agcgttggctacccgtgatattgctgaagagcttggcggcgaatgggctgaccgcttcctcgtgctttacggtatcgccgctccc

gattcgcagcgcatcgccttctatcgccttcttgacgagttcttctgaggggatcaattctctagagctcgctgatcagcctcgact
gtgccttctagttgccagccatctgttgtttgcccctcccccgtgccttccttgaccctggaaggtgccactcccactgtcctttcctaa
taaaatgaggaaattgcatcgcattgtctgagtaggtgtcattctattctggggggtggggtggggcaggacagcaagggggg
aggattgggaagacaatagcaggcatgctggggatgcggtgggctctatggcttctgaggcggaaagaaccagctggggc
tcgaatcaagctgatccggaacccttaatataacttcgtataatgtatgctatacgaagttattaggtccctcgacctgcagcccaa
gctagc**ccaggcaacacctaggacttaTTTTATGCAGCGAGACTGCGAGTTACTACTTCTTAACATCCT**
TTTGTTGCATATTTTCCAGGAGATTGAGAGAGAGGTTAAAAGCTTGATCTCCTGAATTTTATA
CTCTCCCCATTTGAGACAGTTGAGAAATAGGTTAAAGGCATGCTCTCTTGAGTTCCCCATTTG
AGACCGTTGCTACACCGCCAAAACAGAGCATTTTAGATTAGATCTTAAAAATTTAATTCCCC
ACCCTTGCATTCTCAGAGTCAGGCCTTTTAGCAACTCTCACTCATACTTTCAGCCCATTTTCTG
TTTGTTACACTTGCTCATCTTGTCCCAGTCATACCATTGGCTTTCTCCTCTCCTGTTTTTGGTAT
CCCGGTGAGTCATGAAACCAGACAGGTTTCACCACCAATTAAGGCTACCCAGCTCGAGCAT
AGGCTTCACTCTTGCCCAGAAATGCATTTATTCCTCTTTTATGGATATTCTGGAGTCTTTACCT
TGATTTTCATTTAATTTTTTAACCTCAGCTGGGATTCTACTGACCCTCTTAATAGTCCAGATGA
TCTTGACGACTGCTTTGCTGAGAACCGGACGTGAGGTTCAGCAACATCTCTTTTATATCCTTA
GAATACCTTTCAACCCATTTTCATTGATATGCTTATGAGTTAGTAATCAAGCTCAGTTGCCAT
AAGGCTAGTATCCTTCGAACTAGGATCTCTTGTCTCTGGTATCTGCTGATACAACTTTCATAT
GTGTCCAGGACAGTAGTTCTCATACAAAGATAACAGCATGGAAGTAACCGATCCAACTCCTT
TACTGCCTGGTAACTACTGACAGGATGCGTTCCATCATCACAAATGTGATGTACAAGGTCCC
TCAATGGACTAACCTCACCTTAACAGCCTTTTTGTTTGTGACAGTTTTCCACATACACACCCA
AACAATATTATTGGACCTCTTTGTAGGGGTGGTTCCTCCTGGAGTGCTACCCTTGATAGTCCT
TACCCTTCCAATAAAGACTGTTAAAACTCAAATATCATCTCCCCTATGATCTTGCCTTCTGTG
GTCTATGCTTTAAGCTAGAATCCCCTTTCTCTTGGTCCCATACATAGCAGGTTGAATCATAGC
ACTTCTCAGGTGGTTGTCAGTGCTTATTTAAATTATCTTAGCTATTCTGAGCTGCTTGTTGAGT
GTTGTACCTAAGTTCCTAgtgtatttcttgatggctagcgctagcttatcgataccgtcgacggtatcgataagcttga
tatcgaattctaccgggtaggggaggcgcttttcccaaggcagtctggagcatgcgctttagcagccccgctgggcacttggcg
ctacacaagtggcctctggcctcgcacacattccacatccaccggtaggcgccaaccggctccgttctttggtggccccttcgcg
ccaccttctactcctcccctagtcaggaagttcccccccgccccgcagctcgcgtcgtgcaggacgtgacaaatggaagtagca
cgtctcactagtctcgtgcagatggacagcaccgctgagcaatggaagcgggtaggcctttggggcagcggccaatagcagc
tttgctccttcgctttctgggctcagaggctgggaaggggtgggtccgggggcgggctcaggggcgggctcaggggcggg
gcgggcgcccgaaggtcctccggaggcccggcattctgcacgcttcaaaagcgcacgtctgccgcgctgttctcctcttcctcat
ctccgggcctttcgacctgcaggtcctcgccatggatcctgatgatgttgttgattcttctaaatcttttgtgatggaaaacttttcttc
gtaccacgggactaaacctggttatgtagattccattcaaaaaggtatacaaaagccaaaatctggtacacaaggaaattatgac
gatgattggaaagggttttatagtaccgacaataaatacgacgctgcgggatactctgtagataatgaaaacccgctctctggaa
aagctggaggcgtggtcaaagtgacgtatccaggactgacgaaggttctcgcactaaaagtggataatgccgaaactattaag
aaagagttaggtttaagtctcactgaaccgttgatggagcaagtcggaacggaagagtttatcaaaaggttcggtgatggtgct
tcgcgtgtagtgctcagccttcccttcgctgaggggagttctagcgttgaatatattaataactgggaacaggcgaaagcgttaa
gcgtagaacttgagattaattttgaaacccgtggaaaacgtggccaagatgcgatgtatgagtatatggctcaagcctgtgcag
gaaatcgtgtcaggcgatctctttgtgaaggaaccttacttctgtggtgtgacataattggacaaactacctacagagatttaaag
ctctaaggtaaatataaaatttttaagtgtataatgtgttaaactactgattctaattgtttgtgtattttagattccaacctatggaact
gatgaatgggagcagtggtggaatgcagatcctagagctcgctgatcagcctcgactgtgccttctagttgccagccatctgttg
tttgcccctcccccgtgccttccttgaccctggaaggtgccactcccactgtcctttcctaataaaatgaggaaattgcatcgcattgt
ctgagtaggtgtcattctattctggggggtggggtggggcaggacagcaagggggaggattgggaagacaatagcaggca
tgctggggatgcggtgggctctatggcttctgaggcggaaagaaccagctggggctcgagggggggcccggtacccagctt

ttgttccctttagtgagggttaattgcgcgcttggcgtaatcatggtcatagctgtttcctgtgtgaaattgttatccgctcacaattcc
acacaacatacgagccggaagcataaagtgtaaagcctggggtgcctaatgagtgagctaactcacattaattgcgttgcgctc
actgcccgctttccagtcgggaaacctgtcgtgccagctgcattaatgaatcggccaacgcgcggggagaggcggtttgcgta
ttgggcgctcttccgcttcctcgctcactgactcgctgcgctcggtcgttcggctgcggcgagcggtatcagctcactcaaaggc
ggtaatacggttatccacagaatcaggggataacgcaggaaagaacatgtgagcaaaaggccagcaaaaggccaggaacc
gtaaaaaggccgcgttgctggcgtttttccataggctccgcccccctgacgagcatcacaaaaatcgacgctcaagtcagaggt
ggcgaaacccgacaggactataaagataccaggcgtttccccctggaagctccctcgtgcgctctcctgttccgaccctgccgct
taccggatacctgtccgcctttctcccttcgggaagcgtggcgctttctcatagctcacgctgtaggtatctcagttcggtgtaggt
cgttcgctccaagctgggctgtgtgcacgaaccccccgttcagcccgaccgctgcgccttatccggtaactatcgtcttgagtcca
acccggtaagacacgacttatcgccactggcagcagccactggtaacaggattagcagagcgaggtatgtaggcggtgctac
agagttcttgaagtggtggcctaactacggctacactagaagaacagtatttggtatctgcgctctgctgaagccagttaccttcg
gaaaaagagttggtagctcttgatccggcaaacaaaccaccgctggtagcggtggtttttttgtttgcaagcagcagattacgcg
cagaaaaaaaggatctcaagaagatcctttgatcttttctacggggtctgacgctcagtggaacgaaaactcacgttaagggatt
ttggtcatgagattatcaaaaaggatcttcacctagatccttttaaattaaaaatgaagttttaaatcaatctaaagtatatatgagta
aacttggtctgacagttaccaatgcttaatcagtgaggcacctatctcagcgatctgtctatttcgttcatccatagttgcctgactcc
ccgtcgtgtagataactacgatacgggagggcttaccatctggccccagtgctgcaatgataccgcgagacccacgctcaccg
gctccagatttatcagcaataaaccagccagccggaagggccgagcgcagaagtggtcctgcaactttatccgcctccatccag
tctattaattgttgccgggaagctagagtaagtagttcgccagttaatagtttgcgcaacgttgttgccattgctacaggcatcgtg
gtgtcacgctcgtcgtttggtatggcttcattcagctccggttcccaacgatcaaggcgagttacatgatcccccatgttgtgcaaa
aaagcggttagctccttcggtcctccgatcgttgtcagaagtaagttggccgcagtgttatcactcatggttatggcagcactgca
taattctcttactgtcatgccatccgtaagatgcttttctgtgactggtgagtactcaaccaagtcattctgagaatagtgtatgcgg
cgaccgagttgctcttgcccggcgtcaatacgggataataccgcgccacatagcagaactttaaaagtgctcatcattggaaaa
cgttcttcggggcgaaaactctcaaggatcttaccgctgttgagatccagttcgatgtaacccactcgtgcacccaactgatcttca
gcatcttttactttcaccagcgtttctgggtgagcaaaaacaggaaggcaaaatgccgcaaaaaagggaataagggcgacac
ggaaatgttgaatactcatactcttcctttttcaatattattgaagcatttatcagggttattgtctcatgagcggatacatatttgaatg
tatttagaaaaataaacaaataggggttccgcgcacatttccccgaaaagtgccac
