# Supplementary material for: CRISPR/Cas9-mediated knock-in strategy at the Rosa26 locus in cattle fetal fibroblasts
Source: PLoS One. 2022 Nov 28;17(11):e0276811. doi: 10.1371/journal.pone.0276811 (PMC9704577; doi:10.1371/journal.pone.0276811)
Supplement: S3 File — The sequence underlined is the sequence of the 5’ homologous arm targeting the cattle Rosa26. The sequence colored in pink indicates the EF1a Promoter. The sequence colored in green indicates the EGFP gene. The sequence colored in yellow indicates the PGK-NEO-polyA casstte used for cell selection. The sequence underlined and bolded is the 3’ homologous arm targeting the cattle Rosa26. (PDF) [file pone.0276811.s004.pdf]

ctgacgcgccctgtagcggcgcattaagcgcggcgggtgtggtggttacgcgcagcgtgaccgctacacttgccagcgccct
agcgcccgctcctttcgctttcttcccttcctttctcgccacgttcgccggctttccccgtcaagctctaaatcgggggctccctttagg
gttccgatttagtgctttacggcacctcgaccccaaaaaacttgattagggtgatggttcacgtagtgggccatcgccctgataga
cggtttttcgccctttgacgttggagtccacgttctttaatagtggactcttgttccaaactggaacaacactcaaccctatctcggtc
tattcttttgatttataagggattttgccgatttcggcctattggttaaaaaatgagctgatttaacaaaaatttaacgcgaattttaac
aaaatattaacgcttacaatttccattcgccattcaggctgcgcaactgttgggaagggcgatcggtgcgggcctcttcgctatta
cgccagctggcgaaagggggatgtgctgcaaggcgattaagttgggtaacgccagggttttcccagtcacgacgttgtaaaa
cgacggccagtgagcgcgcgtaatacgactcactatagggcgaattggagctccaccgcggGGCGCGccggtagggga
gcggaactctGGTGGGAGGGGGTCCGGCGGATTGGTGGGGGGATGGGTGGCTGAGGTCGTCTGG
CCGGTACCTGGGGGTCGCTTTCCCCGGTGGGAAGAGGGGAGAATAGCGTTTGTTACGCTGAAA
GGGAGAGAGGTGGTCAGAGGCAGGCGGGAGTGCGGCCCGCCTTGCGGCAGCCGAAGGGGGA
GGGAGAAGGGAGCGGAAAAGGCTCGAATCCGGACGGAGCCATTGCTCCTGCAGAGGGAGGA
GCGCTTCCGGCTCTTATCTTGTCACTGATTGGTTGCTGCTGCTCCCGCCGTGTGTGAAAACACAA
ATGGCGTGTTTTGGTTGGAGTAAAGCGCCTGTCAGTTACAGCCTCGGGAGTGCGCAGCCGCCTA
GGGACTCTCGCATTGCCCACTGGGTGGGTGCTTAGGTAGGTAGGGTGGAGAGAGACTTGGATG
AGCAGGCGCGGTCGGCCTCCCACGGGGAGGTAGGGCTGGGGGTGGGAAGGGAGGGTCAGTG
AAAGTGGCTTCGCGCGGGCGTCCTACCACCCACCCCTTCCTTCGGGGGAGTCGGTTTACCCGCC
GCCTGCTTGGCTTCGGCATCTGATTGGCTGCTGAAGCTCAGGGAACGGCCCCTTGTTATTGGCT
CGGGTCCCAAATGAGCGAAACCACTGCGCGGGTCGGCGGGGAGGCGGTCGTTGGTACGATCC
TCCCCGAGACCCAGCGCCGCAGTGTCTGGCCCCGCGCCCCTGCGCAACGTGGCAGGAAGCGC
GCGCTGGAGGTGGGGGCGGACTGCCGGGCCGAGGATTCTGGGTGGTGGCGATTGCGGCTCCG
CCCTGGGCGCCCGCTGCCTGAAGGACAAGACTAGCCCGACCTGCTCCTGGACCCGTGGGGCTG
AAGGGAGGAGTGGGGGTCGGTGCCGCTGGCTTGTGGGTGGGAGGTGCATGTTCTCCAAAAATC
GGCGCGAGCTGCAATCCTGAGGTGGCTGCAGTGGAGGAGGCGGAGAGAAGGCCGCACCCTTC
TCAGCAGGGGGAGGGGAGTGCCGCAATACCTTTATGGGAGTTCTCTGCTGCCTCCTGTCTCCTA
AGGACCGCCCTGGGCCTAGAAGAATCCCTCCCTCCCCCGCGATCTCGTCATCGCCTCCATGTCG
AGTCTCGATTATGGGCGGGATTCTTTTGCCCAGGCTTAACCTTATTCCTGGGCGTTGTCCTGCAG
GGGATCGAGCAGGTATAAGACTTAGAGGACGAACCCAATTTCTTTTATCTTCCCACAGGCTTGA
GTTTGTGTCACAAAATAATTATAATGGggtggtggagtgaaatgaagggCGCGCCATTAATGGTACCtct
agaaaggatctgcgatcgctccggtgcccgtcagtgggcagagcgcacatcgcccacagtccccgagaagttgggggag
gggtcggcaattgaacgggtgcctagagaaggtggcgcggggtaaactgggaaagtgatgtcgtgtactggctccgccttttt
cccgagggtgggggagaaccgtatataagtgcagtagtcgccgtgaacgttctttttcgcaacgggtttgccgccagaacaca
gctgaagcttcgaggggctcgcatctctccttcacgcgcccgccgccctacctgaggccgccatccacgccggttgagtcgcgt
tctgccgcctcccgcctgtggtgcctcctgaactgcgtccgccgtctaggtaagtttaaagctcaggtcgagaccgggcctttgtc
cggcgctcccttggagcctacctagactcagccggctctccacgctttgcctgaccctgcttgctcaactctacgtctttgtttcgtttt
ctgttctgcgccgttacagatccaagctgtgaccggcgcctacgAATTCACCGGTCGCCACCATGGTGAGCAAG
GGCGAGGAGCTGTTCACCGGGGTGGTGCCCATCCTGGTCGAGCTGGACGGCGACGTAAACGG
CCACAAGTTCAGCGTGTCCGGCGAGGGCGAGGGCGATGCCACCTACGGCAAGCTGACCCTGA
AGTTCATCTGCACCACCGGCAAGCTGCCCGTGCCCTGGCCCACCCTCGTGACCACCCTGACCTA
CGGCGTGCAGTGCTTCAGCCGCTACCCCGACCACATGAAGCAGCACGACTTCTTCAAGTCCGC
CATGCCCGAAGGCTACGTCCAGGAGCGCACCATCTTCTTCAAGGACGACGGCAACTACAAGAC
CCGCGCCGAGGTGAAGTTCGAGGGCGACACCCTGGTGAACCGCATCGAGCTGAAGGGCATCG
ACTTCAAGGAGGACGGCAACATCCTGGGGCACAAGCTGGAGTACAACTACAACAGCCACAAC
GTCTATATCATGGCCGACAAGCAGAAGAACGGCATCAAGGTGAACTTCAAGATCCGCCACAAC

ATCGAGGACGGCAGCGTGCAGCTCGCCGACCACTACCAGCAGAACACCCCCATCGGCGACGG
CCCCGTGCTGCTGCCCGACAACCACTACCTGAGCACCCAGTCCGCCCTGAGCAAAGACCCCAA
CGAGAAGCGCGATCACATGGTCCTGCTGGAGTTCGTGACCGCCGCCGGGATCACTCTCGGCAT
GGACGAGCTGTACAAGTAAAGCGGCCGCGACTCTAGATCATAATCAGCCATACCACATTTGTAG
AGGTTTTACTTGCTTTAAAAAACCTCCCACACCTCCCCCTGAACCTGAAACATAAAATGAATGCA
ATTGTTGTTGTTAACTTGTTTATTGCAGCTTATAATGGTTACAAATAAAGCAATAGCATCACAAAT
TTCACAAATAAAGCATTTTTTTCACTGCATTCTAGTTGTGGTTTGTCCAAACTCATCAATGTATCTT
AAGGCggccgctctagaactagtggatccggaacccttaatataacttcgtataatgtatgctatacgaagttattaggtccctc
gacctgcaggaattctaccgggtaggggaggcgcttttcccaaggcagtctggagcatgcgctttagcagccccgctgggcac
ttggcgctacacaagtggcctctggcctcgcacacattccacatccaccggtaggcgccaaccggctccgttctttggtggcccct
tcgcgccaccttctactcctcccctagtcaggaagttcccccccgccccgcagctcgcgtcgtgcaggacgtgacaaatggaagt
agcacgtctcactagtctcgtgcagatggacagcaccgctgagcaatggaagcgggtaggcctttggggcagcggccaatag
cagctttgctccttcgctttctgggctcagaggctgggaaggggtgggtccgggggcgggctcaggggcgggctcaggggc
ggggcgggcgcccgaaggtcctccggaggcccggcattctgcacgcttcaaaagcgcacgtctgccgcgctgttctcctcttcc
tcatctccgggcctttcgacctgcagccaatatgggatcggccattgaacaagatggattgcacgcaggttctccggccgcttgg
gtggagaggctattcggctatgactgggcacaacagacaatcggctgctctgatgccgccgtgttccggctgtcagcgcaggg
gcgcccggttctttttgtcaagaccgacctgtccggtgccctgaatgaactgcaggacgaggcagcgcggctatcgtggctgg
ccacgacgggcgttccttgcgcagctgtgctcgacgttgtcactgaagcgggaagggactggctgctattgggcgaagtgcc
ggggcaggatctcctgtcatctcaccttgctcctgccgagaaagtatccatcatggctgatgcaatgcggcggctgcatacgctt
gatccggctacctgcccattcgaccaccaagcgaaacatcgcatcgagcgagcacgtactcggatggaagccggtcttgtcga
tcaggatgatctggacgaagagcatcaggggctcgcgccagccgaactgttcgccaggctcaaggcgcgcatgcccgacgg
cgatgatctcgtcgtgacccatggcgatgcctgcttgccgaatatcatggtggaaaatggccgcttttctggattcatcgactgtg
gccggctgggtgtggcggaccgctatcaggacatagcgttggctacccgtgatattgctgaagagcttggcggcgaatgggc
tgaccgcttcctcgtgctttacggtatcgccgctcccgattcgcagcgcatcgccttctatcgccttcttgacgagttcttctgaggg
gatcaattctctagagctcgctgatcagcctcgactgtgccttctagttgccagccatctgttgtttgcccctcccccgtgccttccttg
accctggaaggtgccactcccactgtcctttcctaataaaatgaggaaattgcatcgcattgtctgagtaggtgtcattctattctgg
ggggtggggtggggcaggacagcaagggggaggattgggaagacaatagcaggcatgctggggatgcggtgggctcta
tggcttctgaggcggaaagaaccagctggggctcgaatcaagctgatccggaacccttaatataacttcgtataatgtatgctat
acgaagttattaggtccctcgacctgcagcccaagctagc**ccaggcaacacctaggacttaTTTTATGCAGCGAGAC**
TGCGAGTTACTACTTCTTAACATCCTTTTGTTGCATATTTTCCAGGAGATTGAGAGAGAGGTT
AAAAGCTTGATCTCCTGAATTTTATACTCTCCCCATTTGAGACAGTTGAGAAATAGGTTAAA
GGCATGCTCTCTTGAGTTCCCCATTTGAGACCGTTGCTACACCGCCAAAACAGAGCATTTTA
GATTAGATCTTAAAAATTTAATTCCCCACCCTTGCATTCTCAGAGTCAGGCCTTTTAGCAACT
CTCACTCATACTTTCAGCCCATTTTCTGTTTGTTACACTTGCTCATCTTGTCCCAGTCATACCA
TTGGCTTTCTCCTCTCCTGTTTTTGGTATCCCGGTGAGTCATGAAACCAGACAGGTTTCACCA
CCAATTAAGGCTACCCAGCTCGAGCATAGGCTTCACTCTTGCCCAGAAATGCATTTATTCCTC
TTTTATGGATATTCTGGAGTCTTTACCTTGATTTTCATTTAATTTTTTAACCTCAGCTGGGATT
CTACTGACCCTCTTAATAGTCCAGATGATCTTGACGACTGCTTTGCTGAGAACCGGACGTGA
GGTTCAGCAACATCTCTTTTATATCCTTAGAATACCTTTCAACCCATTTTCATTGATATGCTTA
TGAGTTAGTAATCAAGCTCAGTTGCCATAAGGCTAGTATCCTTCGAACTAGGATCTCTTGTCT
CTGGTATCTGCTGATACAACTTTCATATGTGTCCAGGACAGTAGTTCTCATACAAAGATAAC
AGCATGGAAGTAACCGATCCAACTCCTTTACTGCCTGGTAACTACTGACAGGATGCGTTCCA
TCATCACAAATGTGATGTACAAGGTCCCTCAATGGACTAACCTCACCTTAACAGCCTTTTTGT
TTGTGACAGTTTTCCACATACACACCCAAACAATATTATTGGACCTCTTTGTAGGGGTGGTTC

CTCCTGGAGTGCTACCCTTGATAGTCCTTACCCTTCCAATAAAGACTGTTAAAACTCAAATAT
CATCTCCCCTATGATCTTGCCTTCTGTGGTCTATGCTTTAAGCTAGAATCCCCTTTCTCTTGGT
CCCATACATAGCAGGTTGAATCATAGCACTTCTCAGGTGGTTGTCAGTGCTTATTTAAATTAT
CTTAGCTATTCTGAGCTGCTTGTTGAGTGTTGTACCTAAGTTCCTAgtgtatttcttgatggctagcgc
tagcttatcgataccgtcgacggtatcgataagcttgatatcgaattctaccgggtaggggaggcgcttttcccaaggcagtctg
gagcatgcgctttagcagccccgctgggcacttggcgctacacaagtggcctctggcctcgcacacattccacatccaccggta
ggcgccaaccggctccgttctttggtggccccttcgcgccaccttctactcctcccctagtcaggaagttcccccccgccccgcag
ctcgcgtcgtgcaggacgtgacaaatggaagtagcacgtctcactagtctcgtgcagatggacagcaccgctgagcaatgga
agcgggtaggcctttggggcagcggccaatagcagctttgctccttcgctttctgggctcagaggctgggaaggggtgggtcc
gggggcgggctcaggggcgggctcaggggcggggcgggcgcccgaaggtcctccggaggcccggcattctgcacgcttc
aaaagcgcacgtctgccgcgctgttctcctcttcctcatctccgggcctttcgacctgcaggtcctcgccatggatcctgatgatgtt
gttgattcttctaaatcttttgtgatggaaaacttttcttcgtaccacgggactaaacctggttatgtagattccattcaaaaaggtata
caaaagccaaaatctggtacacaaggaaattatgacgatgattggaaagggttttatagtaccgacaataaatacgacgctgcg
ggatactctgtagataatgaaaacccgctctctggaaaagctggaggcgtggtcaaagtgacgtatccaggactgacgaaggt
tctcgcactaaaagtggataatgccgaaactattaagaaagagttaggtttaagtctcactgaaccgttgatggagcaagtcgg
aacggaagagtttatcaaaaggttcggtgatggtgcttcgcgtgtagtgctcagccttcccttcgctgaggggagttctagcgtt
gaatatattaataactgggaacaggcgaaagcgttaagcgtagaacttgagattaattttgaaacccgtggaaaacgtggcca
agatgcgatgtatgagtatatggctcaagcctgtgcaggaaatcgtgtcaggcgatctctttgtgaaggaaccttacttctgtggt
gtgacataattggacaaactacctacagagatttaaagctctaaggtaaatataaaatttttaagtgtataatgtgttaaactactga
ttctaattgtttgtgtattttagattccaacctatggaactgatgaatgggagcagtggtggaatgcagatcctagagctcgctgat
cagcctcgactgtgccttctagttgccagccatctgttgtttgcccctcccccgtgccttccttgaccctggaaggtgccactcccac
tgtcctttcctaataaaatgaggaaattgcatcgcattgtctgagtaggtgtcattctattctggggggtggggtggggcaggac
agcaagggggaggattgggaagacaatagcaggcatgctggggatgcggtgggctctatggcttctgaggcggaaagaa
ccagctggggctcgagggggggcccggtacccagcttttgttccctttagtgagggttaattgcgcgcttggcgtaatcatggt
catagctgtttcctgtgtgaaattgttatccgctcacaattccacacaacatacgagccggaagcataaagtgtaaagcctggggt
gcctaatgagtgagctaactcacattaattgcgttgcgctcactgcccgctttccagtcgggaaacctgtcgtgccagctgcatta
atgaatcggccaacgcgcggggagaggcggtttgcgtattgggcgctcttccgcttcctcgctcactgactcgctgcgctcggt
cgttcggctgcggcgagcggtatcagctcactcaaaggcggtaatacggttatccacagaatcaggggataacgcaggaaag
aacatgtgagcaaaaggccagcaaaaggccaggaaccgtaaaaaggccgcgttgctggcgtttttccataggctccgccccc
ctgacgagcatcacaaaaatcgacgctcaagtcagaggtggcgaaacccgacaggactataaagataccaggcgtttccccct
ggaagctccctcgtgcgctctcctgttccgaccctgccgcttaccggatacctgtccgcctttctcccttcgggaagcgtggcgctt
tctcatagctcacgctgtaggtatctcagttcggtgtaggtcgttcgctccaagctgggctgtgtgcacgaaccccccgttcagcc
cgaccgctgcgccttatccggtaactatcgtcttgagtccaacccggtaagacacgacttatcgccactggcagcagccactggt
aacaggattagcagagcgaggtatgtaggcggtgctacagagttcttgaagtggtggcctaactacggctacactagaagaa
cagtatttggtatctgcgctctgctgaagccagttaccttcggaaaaagagttggtagctcttgatccggcaaacaaaccaccgct
ggtagcggtggtttttttgtttgcaagcagcagattacgcgcagaaaaaaaggatctcaagaagatcctttgatcttttctacggg
gtctgacgctcagtggaacgaaaactcacgttaagggattttggtcatgagattatcaaaaaggatcttcacctagatccttttaaa
ttaaaaatgaagttttaaatcaatctaaagtatatatgagtaaacttggtctgacagttaccaatgcttaatcagtgaggcacctatc
tcagcgatctgtctatttcgttcatccatagttgcctgactccccgtcgtgtagataactacgatacgggagggcttaccatctggc
cccagtgctgcaatgataccgcgagacccacgctcaccggctccagatttatcagcaataaaccagccagccggaagggccg
agcgcagaagtggtcctgcaactttatccgcctccatccagtctattaattgttgccgggaagctagagtaagtagttcgccagtt
aatagtttgcgcaacgttgttgccattgctacaggcatcgtggtgtcacgctcgtcgtttggtatggcttcattcagctccggttccc
aacgatcaaggcgagttacatgatcccccatgttgtgcaaaaaagcggttagctccttcggtcctccgatcgttgtcagaagtaa
gttggccgcagtgttatcactcatggttatggcagcactgcataattctcttactgtcatgccatccgtaagatgcttttctgtgactg

gtgagtactcaaccaagtcattctgagaatagtgtatgcggcgaccgagttgctcttgcccggcgtcaatacgggataataccg
cgccacatagcagaactttaaaagtgctcatcattggaaaacgttcttcggggcgaaaactctcaaggatcttaccgctgttgag
atccagttcgatgtaacccactcgtgcacccaactgatcttcagcatcttttactttcaccagcgtttctgggtgagcaaaaacagg
aaggcaaaatgccgcaaaaaagggaataagggcgacacggaaatgttgaatactcatactcttcctttttcaatattattgaagc
atttatcagggttattgtctcatgagcggatacatatttgaatgtatttagaaaaataaacaaataggggttccgcgcacatttcccc
gaaaagtgccac
