# Supplementary material for: CRISPR/Cas9-mediated knock-in strategy at the Rosa26 locus in cattle fetal fibroblasts
Source: PLoS One. 2022 Nov 28;17(11):e0276811. doi: 10.1371/journal.pone.0276811 (PMC9704577; doi:10.1371/journal.pone.0276811)
Supplement: S4 File — (PDF) [file pone.0276811.s005.pdf]

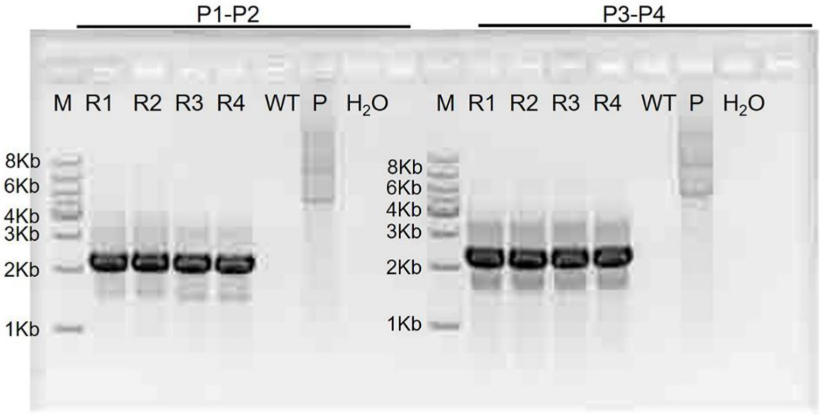

Raw images of S1 Fig.

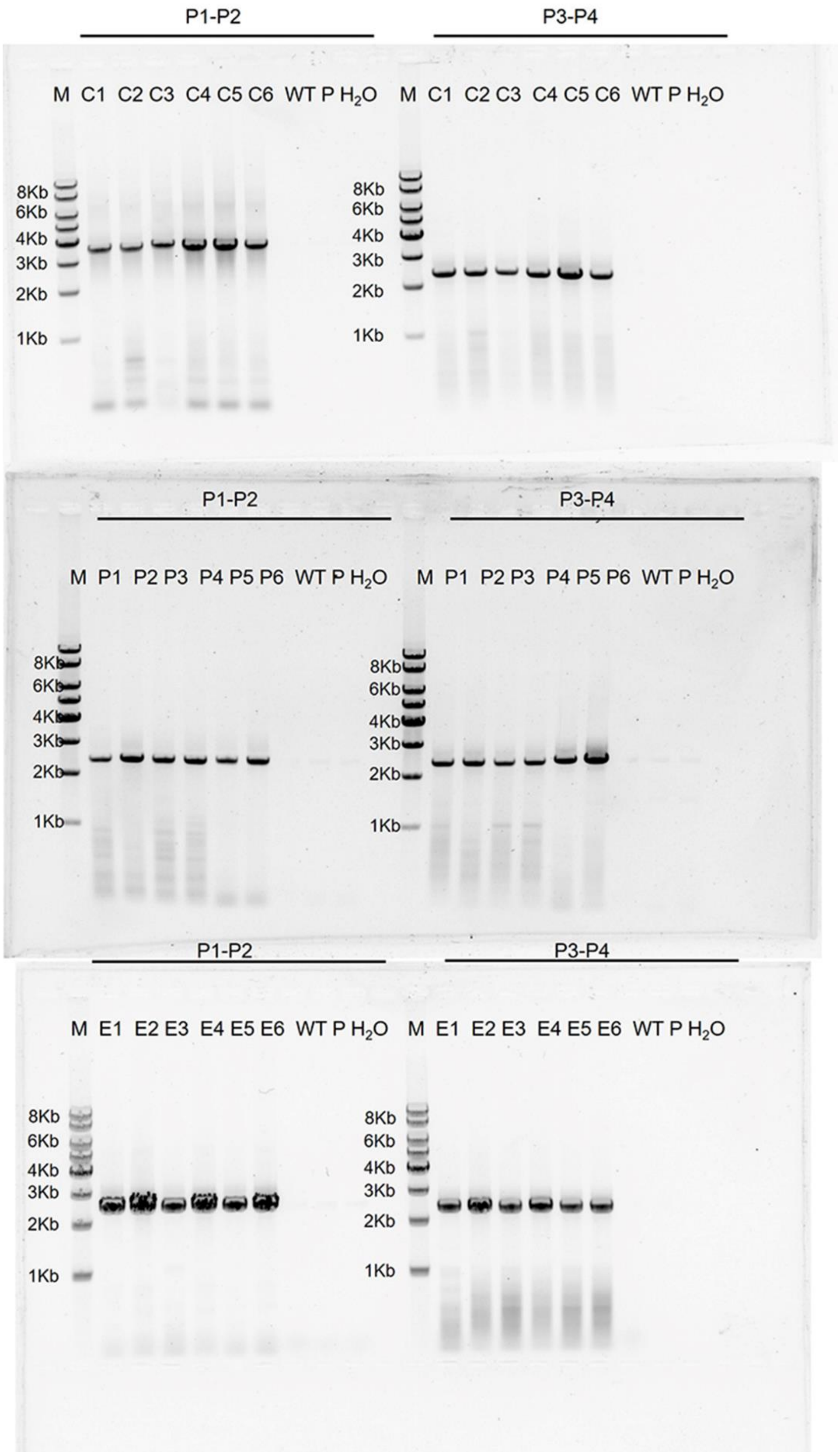

Raw images of Fig 2B.

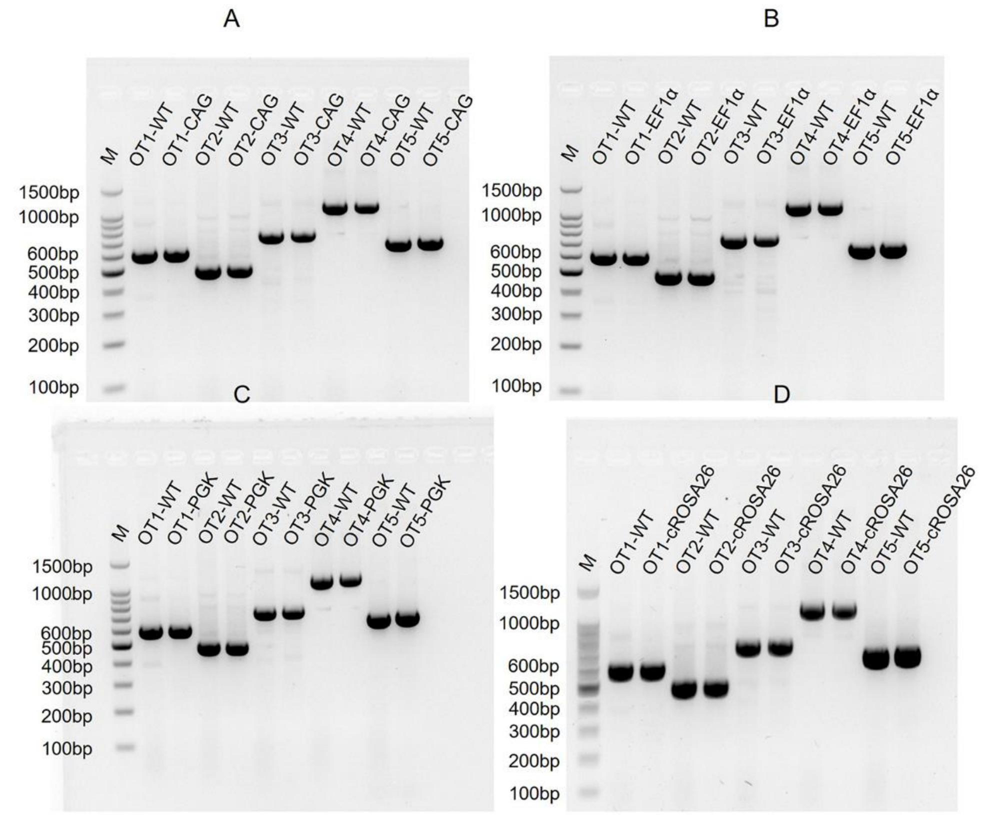

Raw images of Fig 3.

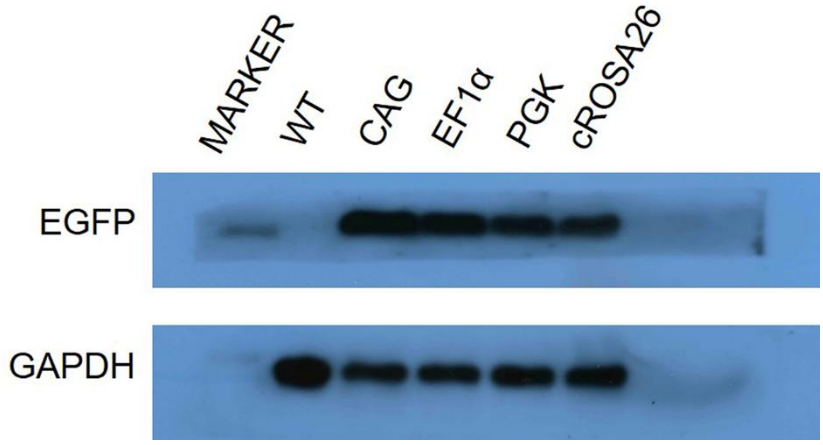

Raw images of Fig 4B.

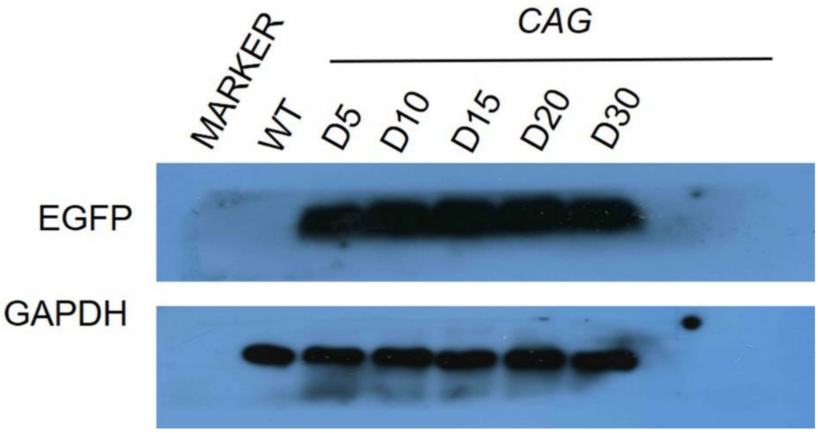

Raw images of Fig 4D.

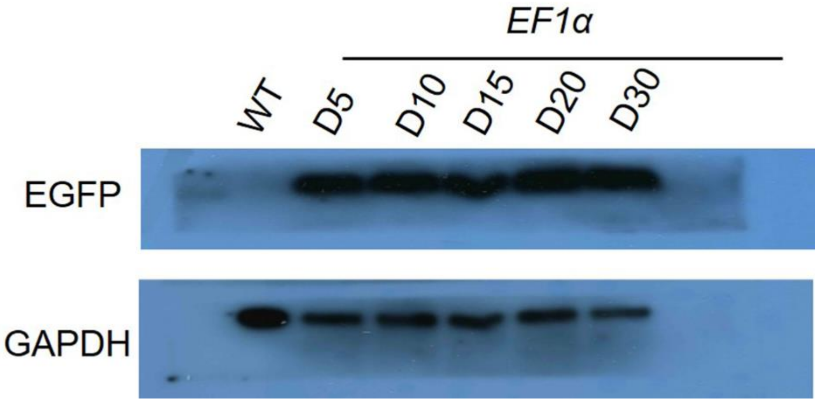

Raw images of Fig 4E.

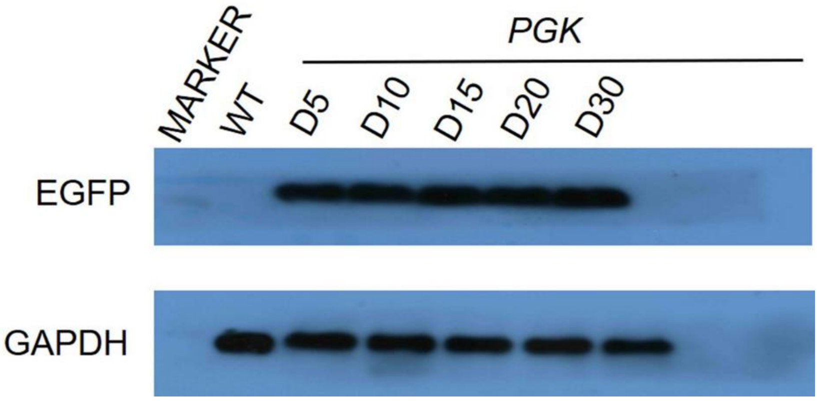

Raw images of Fig 4F.

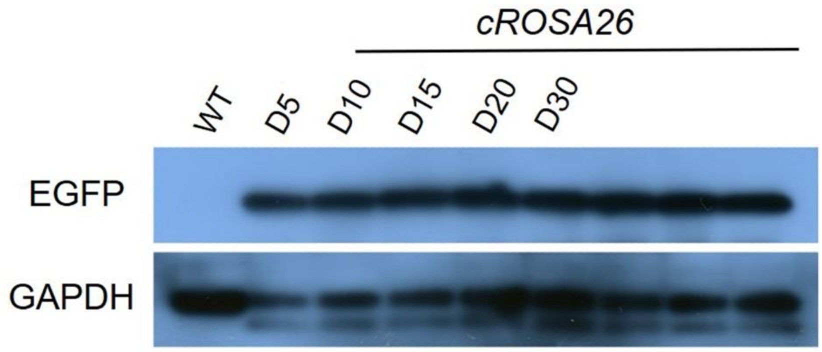

Raw images of Fig 4G.
