# Supplementary material for: CRISPR/Cas9-mediated knock-in strategy at the Rosa26 locus in cattle fetal fibroblasts
Source: PLoS One. 2022 Nov 28;17(11):e0276811. doi: 10.1371/journal.pone.0276811 (PMC9704577; doi:10.1371/journal.pone.0276811)
Supplement: S1 Raw images — (PDF) [file pone.0276811.s006.pdf]

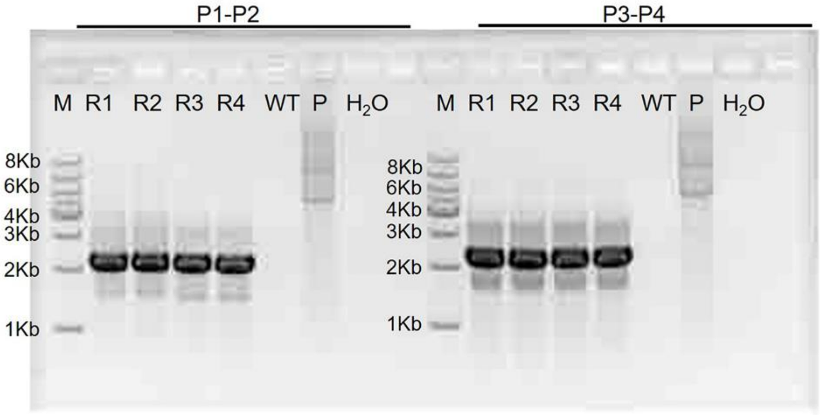

S1_raw_images of S1 Fig 1.

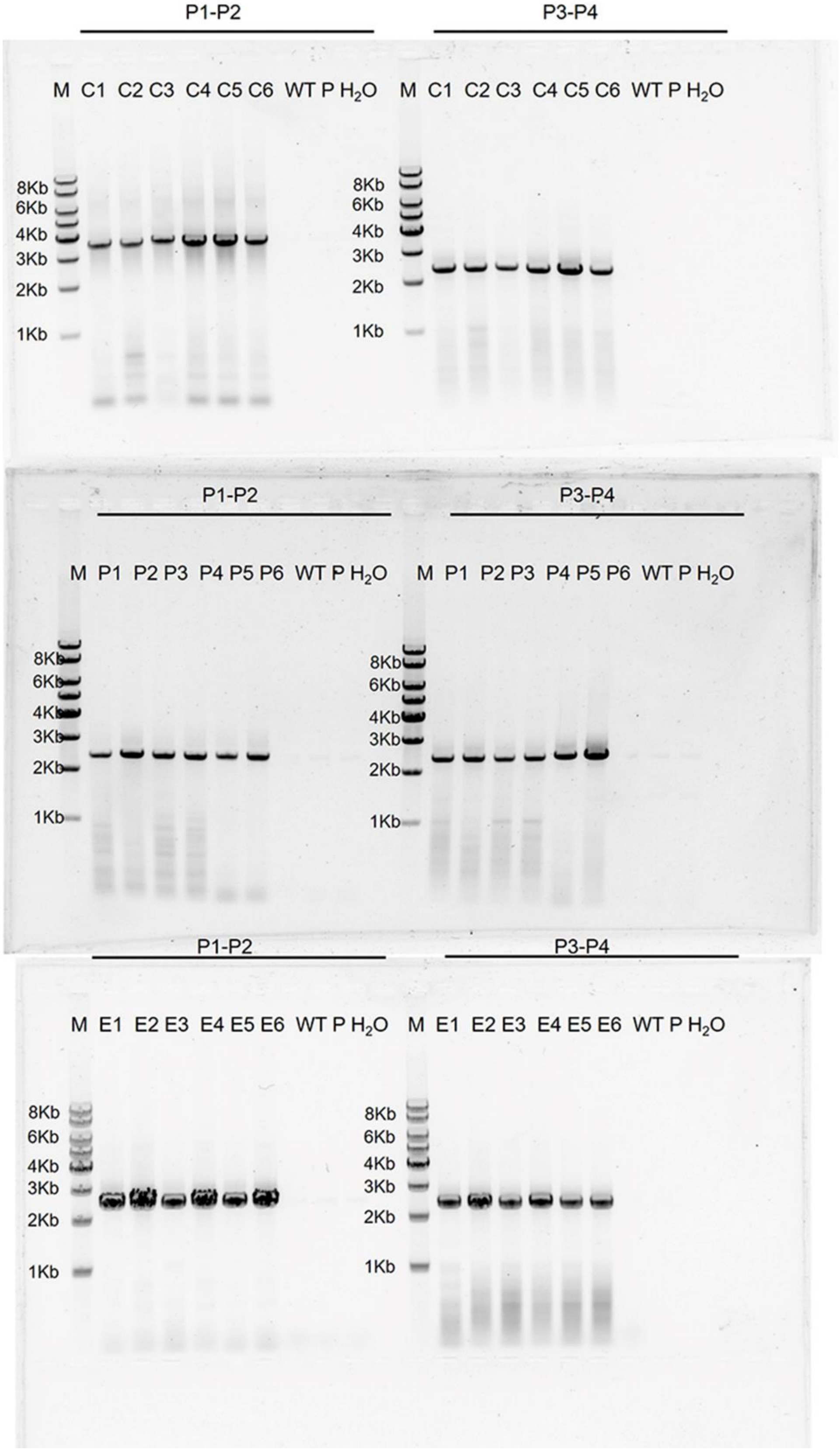

S1_raw_images of Fig. 2B.

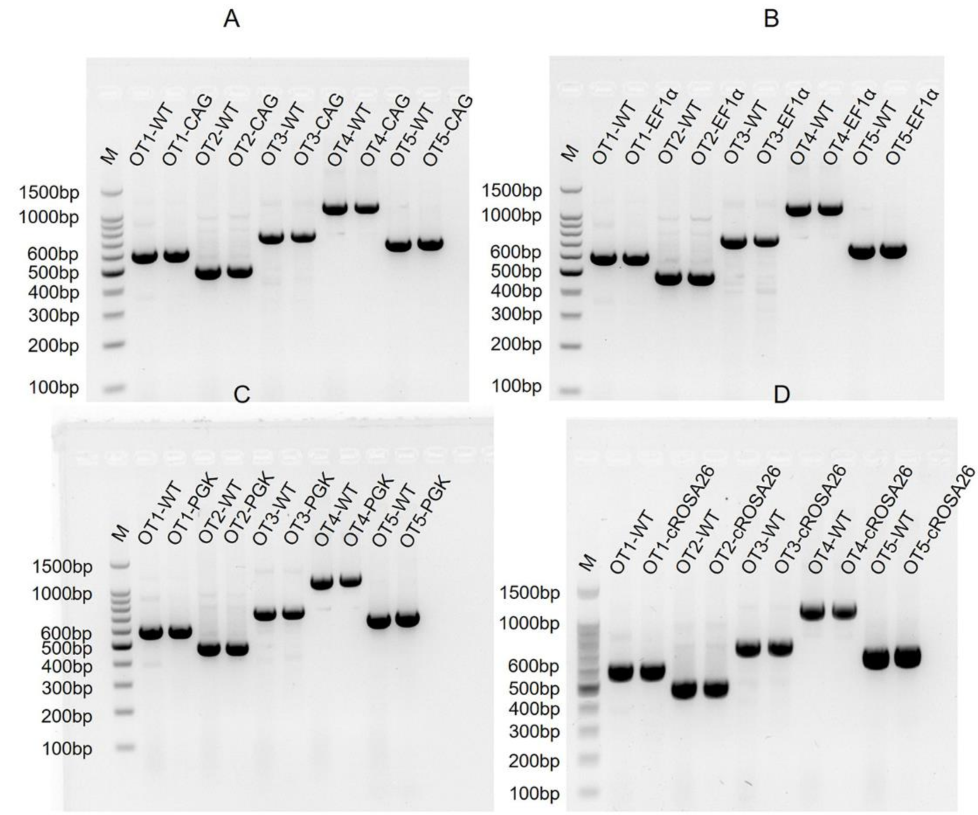

S1_raw_images of Fig. 3.

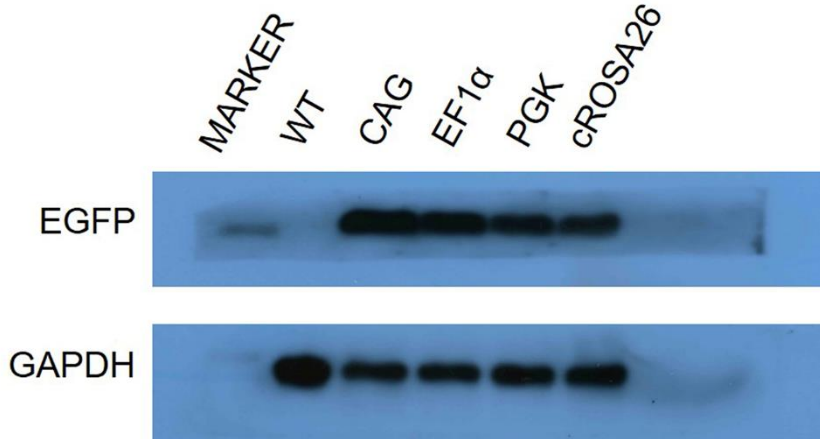

S1_raw_images of Fig. 4B.

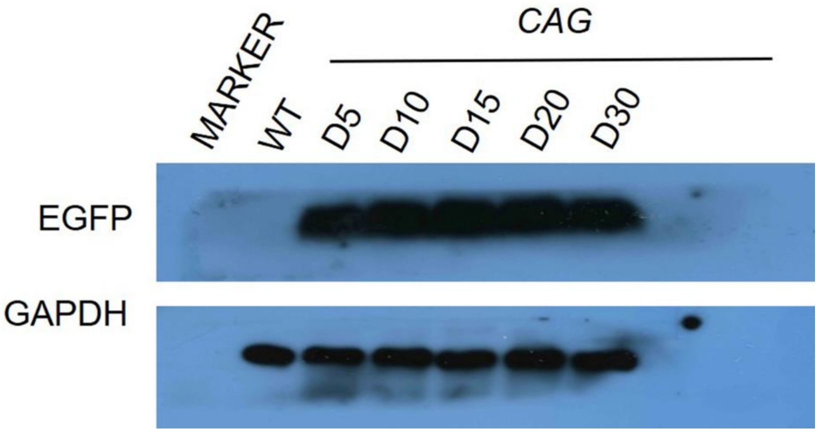

S1_raw_images of Fig. 4D.

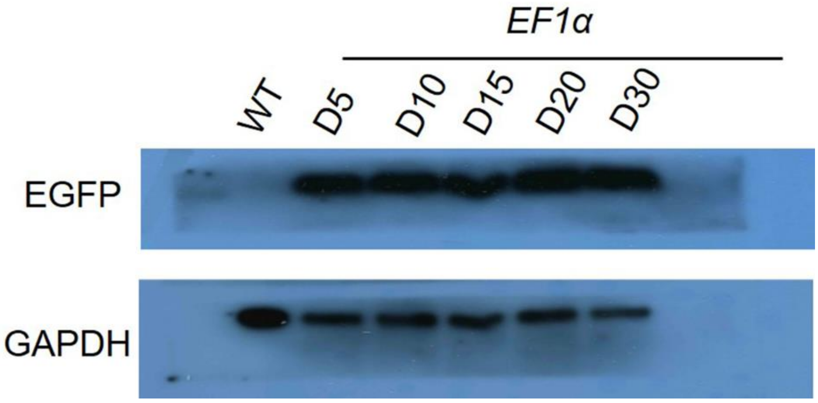

S1_raw_images of Fig. 4E.

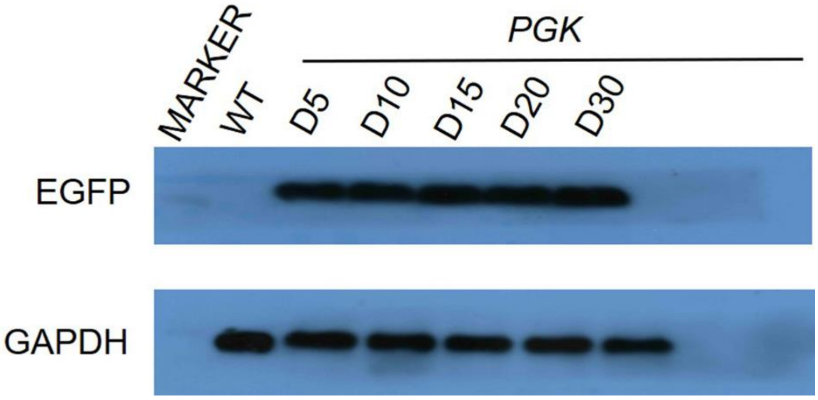

S1_raw_images of Fig. 4F.

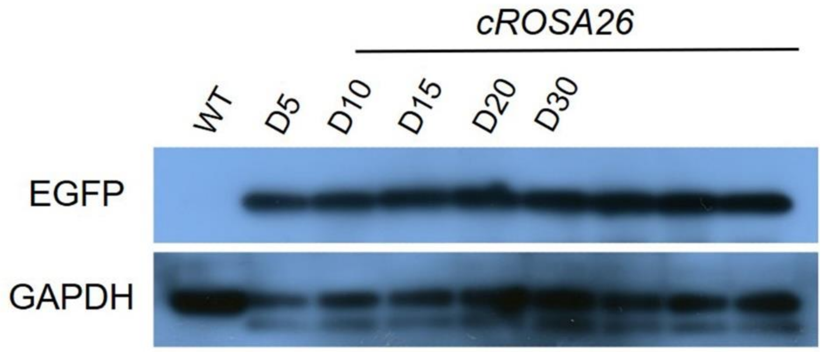

S1_raw_images of Fig. 4G.
